# NMR evidence for residue-based LFER relationship in two-state folding-unfolding equilibrium of the spectrin SH3 domain in acidic solutions

**DOI:** 10.2142/biophysico.bppb-v23.0011

**Published:** 2026-03-05

**Authors:** Seiichiro Hayashi, Daisuke Fujinami, Daisuke Kohda

**Affiliations:** 1 Institute for Molecular Science, National Institutes of Natural Sciences, Okazaki, Aichi 444-8787, Japan; 2 Graduate School of Integrated Pharmaceutical and Nutritional Sciences, University of Shizuoka, Shizuoka 422-8526, Japan; 3 Medical Institute of Bioregulation, Kyushu University, Fukuoka 812-8582, Japan

**Keywords:** linear free energy relationship, ϕ-value analysis, protein refolding, transition state ensemble, two-state exchange

## Abstract

The conformational change of a protein molecule is described by the single equilibrium constant *K* and the single rate constants, *k* (forward) and *k’* (reverse). The underlying assumption is that all amino acid residues undergo state changes with perfect cooperativity. However, residue-specific measurements such as NMR often show residue-to-residue variations in these constants, indicating that proteins are not fully cooperative. Theoretical considerations based on “the consistency principle of protein folding” demonstrated that the linear free energy relationship (LFER) observed in residue-specific log *k* vs. log *K* plots is the physicochemical basis for smooth conformational changes of protein molecules. The residue-based LFER was found in many protein-related phenomena, but the structural changes have been limited to relatively small ones, such as fluctuations in the folded state and the coupled binding and folding of intrinsically disordered proteins. Here, we applied NMR to the two-state exchange equilibrium of the spectrin SH3 domain in acidic solutions and determined residue-specific equilibrium and rate constants. The data points obtained from the wild-type and its two single amino acid mutants were aligned on a single line in the log *k* vs. log *K* plot, indicating that residue-based LFER applies to large structural changes between the unfolded and folded states of the SH3 domain. The mutation-induced shifts in the distribution ranges of residue-specific equilibrium and rate constants are useful for establishing residue-based LFERs for all kinds of protein structural changes, including the refolding from a fully unfolded state, caused by the removal of a denaturant.

## Significance

The ϕ-value analysis has been widely used to analyze the transition state in protein refolding reactions. The ϕ value is the extent of native structure formation around a mutated residue in the transition state. The residue-based LFER and its generalized QFER, where Q stands for quadratic, also provide residue-specific information φ (similar to ϕ). Here, we demonstrated that the use of amino acid mutations expanded the applicability of residue-based LFER/QFER. Furthermore, we clarified the relationship between residue-based LFER/QFER and the ϕ analysis and revealed that both ϕ and φ provided equivalent structural information about the transition state.

## Introduction

**Figure 1:**
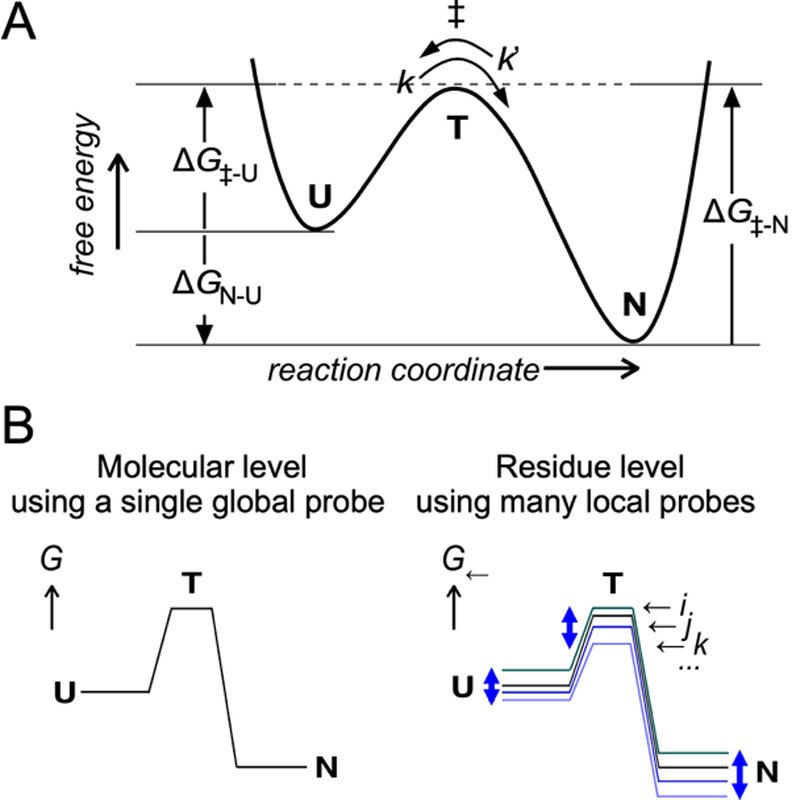
Molecular- and residue-level energy diagrams. (A) The energetic basis of protein structural changes. Note that the sign of Δ*G*_N-U_ is negative because state N is more stable than state U. The equilibrium and rate constants can be converted into the corresponding free energy differences using the following equations: Δ*G*_N-U_=–*RT* ln *K*, Δ*G*_‡-U_=–*RT* ln *k*+*RT* ln *A*, and Δ*G*_‡-N_=–*RT* ln *k’*+*RT* ln *A*, where *R* is the gas constant, *T* is the absolute temperature, and *A* is the pre-exponential factor of the Arrhenius formula. To emphasize the mixed state of a huge number of different conformations, USE (unfolded state ensemble) and TSE (transition state ensemble) are used, instead of U and T. (B) Comparison of the free energy level diagrams at the molecular and residue levels. The symbols ←*i*, ←*j*, and ←*k* represent measurements using different local probes.

The analysis of transition states in the structural changes of protein molecules is an important topic in protein science (Fig. 1A) [1,2]. To follow protein conformational changes, the fluorescence of tryptophan residues is often monitored due to its high sensitivity and excellent time resolution [3,4]. Although tryptophan fluorescence is inherently localized information reflecting the surrounding microenvironment, the equilibrium and rate constants obtained from fluorescence measurements are typically treated as single physical quantities representing the entire protein molecule. Behind this convention is the implicit assumption that the amino acid residues and atoms that constitute a protein molecule act in perfect concert during structural changes. In practice, however, experimental determinations of the residue-specific equilibrium constant *K* and rate constants *k* (forward) and *k’* (reverse) often yield different values. The free energy levels of the three states, unfolded state (U), transition state (T), and native state (N), are single physical quantities (left, Fig. 1B), but at the residue level using local probes, the observed free energy values differ, reflecting the microenvironments around the probe positions (right, Fig. 1B). To avoid confusion, local probe observations are denoted by a special symbol, ← [5]. Following this notation, we use symbols such as *G*_←_ and Δ*G*_←_. Similarly, the correlation between log *k* and log *K* on a residue-by-residue basis is expressed as log *k*_←_ vs. log *K*_←_.

In almost all cases, assuming measurement error, the measurements are reduced to a single value by global fitting or averaging. However, we suggest an alternative interpretation: the variation in these constants is due to a decrease in the overall cooperativity in the structural changes of protein molecules. Previously, we conducted a literature search to collect the residue-specific *K*, *k*, and *k’* values of protein structural changes from residue-level measurements and constructed log *k*_←_ vs. log *K*_←_ plots and log *k’*_←_ vs. log *K*_←_ plots [6]. In more than half of the cases, we found linear relationships in these log-log plots. In other unconfirmed cases, it is possible that the measurement errors (*i.e.*, the systematic error, which is an inherent bias in the measurement method, and the random error associated with the measurement) were not small enough, or the assumption of the two-state exchange equilibrium was invalid. The linear relationship in the log-log plots represents the linear correlation between the free energy change from state U to state N and the free energy change from one of the two ground states to the transition state in a two-state exchange system (Fig. 1A). These linear correlations are called linear free energy relationships (LFERs). LFERs have been found empirically in many natural phenomena, such as chemical and biochemical reactions [7–10], but the LFER we discovered is a special type obtained by monitoring *multiple* amino acid residues in a *single* polypeptide chain under a *single* condition. Therefore, we named it residue-based LFER (rbLFER) [11]. We hypothesized that rbLFER is related to the fundamental physicochemical properties of foldable polypeptide chains. In 1983, Nobuhiro Gō proposed the “consistency principle” theory of protein folding [12]. We demonstrated that rbLFER can be mathematically derived from the consistency principle of protein folding [5].

The ϕ-value analysis using single amino acid mutations, developed by Alan Fersht, has been widely used to analyze the transition state in the refolding reactions of many small proteins [13–17]. The ϕ value represents the local fraction of the native state around the side chain of a mutated residue in the transition state ensemble and is defined by the following formula,

(1) $$\phi =\frac{{\log k}_{wt}-{\log k}_{mut}}{{\log K}_{wt}-{\log K}_{mut}}=\frac{\Delta logk}{\Delta logK}$$

where *wt* and *mut* represent the wild-type protein and its single amino acid mutant, respectively. Here, the mutant values are subtracted from the wild-type values. This is because introducing a mutation is expected to decrease the rate of structural formation *k* and the equilibrium constant *K*. As a result, both the numerator and denominator of Eq. 1 are expected to be positive. Ideally, the ϕ value is either 0 or 1, but experimentally it is usually between 0 and 1. The premise of the ϕ-value analysis is that an amino acid residue adopts only two states during the structural change: state U and state N; that is, no non-native states exist [16]. Another assumption is that amino acid mutations do not significantly change the free energy level of state U [16]. If these assumptions are violated, then the ϕ value becomes negative or greater than one.

**Figure 2:**
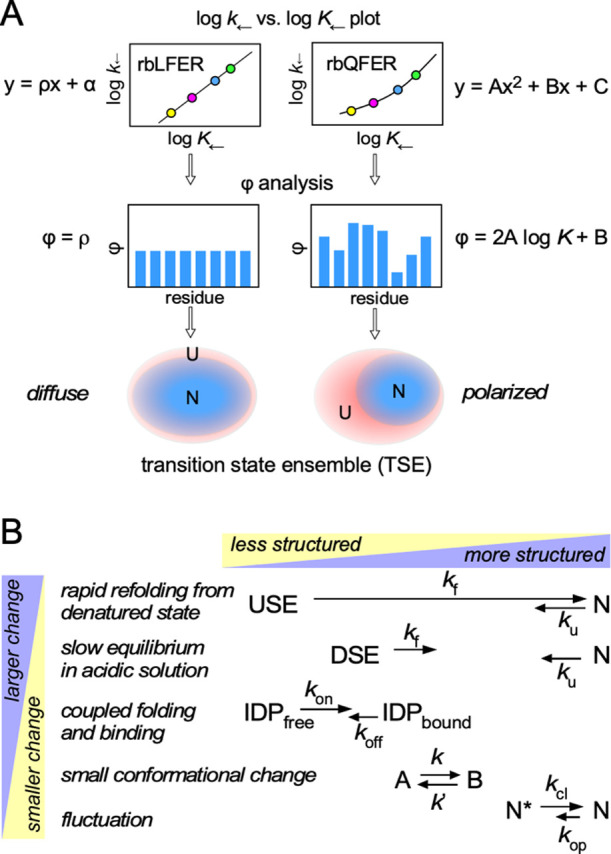
Residue-level transition state analysis. (A) The data points in the log *k*_←_ vs. log *K*_←_ plot show a linear relationship (LFER) and a parabolic relationship (QFER, where Q stands for quadratic). The symbol φ represents the local fraction of state N around the residue of interest in the transition state ensemble (TSE). In residue-based LFER, all φ values are equal in the TSE, and the distribution of state N in the TSE is uniform. In contrast, in residue-based QFER, φ values vary from residue to residue, and the distribution of state N in the TSE is biased. The former TSE state is called “diffuse”, and the latter TSE state is called “polarized” [10]. Note that if the measurement error is large, rbQFER must be analyzed as rbLFER. (B) Classification of protein structural changes according to their magnitude. In the USE state induced by denaturants, protein molecules exist in an almost fully unfolded state with a vast number of different conformations. In the DSE (denatured state ensemble) state, protein molecules exist in the form of molten globules [21,22]. The IDP folds upon binding to target proteins. Some peptides/proteins exist in a two-state slow exchange with small conformational changes. Fully folded proteins undergo fluctuations that loosen their structures, causing labile protons to exchange with the solvent water.

In the theoretical study, we found that the residue-based LFER is a special case of a more general residue-based QFER, where Q stands for quadratic [5]. The residue-based LFER/QFER can provide similar information to the ϕ value: the fraction of state N around a local probe (e.g., amide ^1^H nucleus) in the transition state ensemble (Fig. 2A). Here, we intend to use a different symbol, φ, to distinguish it from the ϕ value. The φ and ϕ values emphasize the similarities in the physical meanings and the differences in the experimental methodologies and their applicable target systems.

Although we found the residue-based LFER in many protein-related phenomena [6], the structural changes have been limited to relatively small ones, such as coupled binding and folding of IDPs (intrinsically disordered proteins), conformational changes in small parts of protein molecules, and fluctuations in the folded state (Fig. 2B). The remaining challenge is to experimentally prove that rbLFER/QFER applies to the two-state exchange of protein molecules with large structural changes. In this study, we determined the residue-specific equilibrium and rate constants in the two-state exchange equilibrium in acidic solution between the unfolded and folded states of the chicken α-spectrin SH3 domain (62 residues) and its two single amino acid mutants. We discovered that data points for each protein did not align well in a log *k*_←_ vs. log *K*_←_ plot, but when the three proteins were combined, the data points aligned neatly on a single line. This demonstrates that combining local probe observations with single amino acid mutations has expanded the scope of residue-based LFER/QFER, making it applicable to larger structural changes in protein molecules than ever before. As rbLFER can be derived from the consistency principle of protein folding [12], the combined method established in this study is expected to serve as a powerful experimental approach for elucidating the operation of this principle during the refolding of proteins from a fully unfolded state under physiological conditions.

## Materials and methods

### Protein purification and NMR sample preparation

The DNA fragment encoding the chicken α-spectrin SH3 domain (62 residues) was subcloned into the pGEX-6P-1 vector (Cytiva). Single point mutations, L33A and V46A, were introduced to the spectrin SH3 sequence by inverse PCR-based site-directed mutagenesis. The transformed *Escherichia coli* BL21 (DE3) cells (Novagen) were grown at 310 K in M9 medium, supplemented with ^15^NH_4_Cl and 100 mg L^–1^ ampicillin. When the OD_600_ reached 0.4–0.6, the culture was cooled to 293 K, supplemented with 1 mM IPTG, and incubated overnight. After centrifugation, the cell pellets were resuspended in lysis buffer (20 mM Tris-HCl, pH 8.5, 150 mM NaCl, 1 mM DTT) and disrupted by sonication. After centrifugation, the cell lysate was gently stirred with glutathione Sepharose 4B resin (Cytiva), pre-equilibrated with lysis buffer. The GST-fusion protein was cleaved on-resin with 3C protease. The cleaved protein was further separated by gel filtration chromatography with Superdex75 10/300 GL (Cytiva) and reverse-phase HPLC with COSMOSIL 5C18-AR-300 (Nacalai Tesque). The purified SH3 proteins, wild-type, L33A, and V46A, were dissolved at final concentrations of 1.0 mM, 2.2 mM, and 1.8 mM in water containing 10% (v/v) ^2^H_2_O, and the pH was adjusted to 2.4, 3.6, and 2.4 with 0.2 M NaOH, respectively. The pH of the protein solutions was adjusted to shift the equilibrium between DSE and N to an approximate 1:1 ratio for optimal EXSY measurement and data analysis. The ϕ values of each amino acid mutation determined in the previous studies were almost equal at pH 2.5 and 3.5 [18]. Given this, the effect of pH is considered minimal in the NMR experiments performed under the acidic conditions of pH 2.4 and 3.6.

The unfolding and refolding kinetics of the spectrin SH3 domain, monitored by tryptophan fluorescence and circular dichroism at pH 3.5, provided evidence for a folding mechanism consistent with a two-state process without the accumulation of intermediates [19]. In the ^1^H-^15^N HSQC spectrum of the spectrin SH3 domain at pH 2.2, two sets of cross peaks from amide groups were observed, indicating that the SH3 domain exists in two states in acidic solutions [20]. The chemical shift dispersion of the cross peaks suggested that one of the two states is similar to the native globular state at neutral pH, while the other is unstructured. Acid-denatured proteins are known to exist in a molten globule state, adopting compact protein structures with secondary structure contents similar to those of the native folded proteins, but with poorly defined tertiary structures [21,22]. The structural properties of the spectrin SH3 protein in acidic solutions ranging from pH 2 to 4 were previously reported [19,20,23–28]. The two-state exchange under acidic conditions is exceptionally slow: with its exchange rate *k*_ex_ (=*k*_f_+*k*_u_) in the sub-second regime, the spectrin SH3 domain is well suited for EXSY NMR measurements. Slow exchange rates under acidic conditions were also reported for other SH3 domains, including src SH3 and drk NSH3 [29,30]. As suitable model proteins for protein folding, SH3 domains have been extensively studied over the past 30 years ([2,13] and references therein).

### NMR spectroscopy

NMR spectra were recorded on an Avance 600 spectrometer equipped with a TXI cryoprobe (Bruker) at 298 K. Each 2D spectrum was recorded as a complex data matrix composed of 256×1024 points with spectral widths of 1885 and 7184 Hz in the *t*_1_ and *t*_2_ dimensions, respectively. ^1^H-^15^N HSQC*i* (*i*=1, 2, 3) spectra (Supplementary Fig. S1A, S1B, and S1C) and ^1^H-^15^N EXSY spectra (Supplementary Fig. S1D) were recorded using the pulse sequences described previously [31–33]. Sixteen scans per FID were collected. To investigate the dependence of log *K*_←_ on the relaxation delay (d1), a preliminary experiment was performed using ^1^H-^15^N HSQC spectra. The log *K*_←_ values of some representative residues reached a plateau at d1=4 s. Thus, d1 was set to 5.0 s. A set of HSQC*i* experiments (*i*=1, 2, 3) was repeated four times to reduce the measurement error. The measurement time of a single HSQC*i* spectrum was about 12 h. The EXSY experiment with a mixing time of 0.4 s was repeated four times. To check the linearity of the EXSY-Π plot, the additional EXSY spectra were measured with mixing times of 0.1, 0.15, 0.2, 0.3, and 0.6 s. The measurement time of a single EXSY spectrum was about 12 h. Detailed measurement setting conditions were described in the previous study [34].

### NMR data analysis

All NMR data were processed with nmrPipe and displayed with nmrDraw and POKY [35,36]. A Gaussian window function was applied with options, “-fn GM -g2 5 -c 1.0”, to the F2 (^1^H) dimension of the HSQC*i* spectra, whereas a shifted sine-bell window function was applied with options, “-fn SP -off 0.45 -end 0.98 -pow 1 -c 1.0”, to the F2 (^1^H) dimension of the EXSY spectra. The FID data were subsequently zero-filled and Fourier transformed, and the 6.5–10.5 ppm region of the ^1^H dimension was extracted. After transpose, the interferograms were apodized by a Gaussian window function with options, “-fn GM -g2 5 -c 0.5”, to the F1 (^15^N) dimension of the HSQC*i* spectra, and a shifted sine-bell window function with options, “-fn SP -off 0.5 -end 0.98 -pow 1 -c 0.5”, to the F1 (^15^N) dimension of the EXSY spectra, and then zero-filled twice to 1024 complex points prior to FT. Although the second zero-filling does not provide any additional information, the additional zero-filling increases the digital resolution and thus improves the definition of peak shapes in the projection spectra on the ^15^N axis. For reprocessing spectra with double zero-filling, the NMRbox VM was accessed remotely through a virtual network computing (VNC) client [37]. Three-dimensional HNCA and HNCOCA spectra of the ^15^N-, ^13^C-labeled wild-type and mutant SH3 proteins were measured to assign the backbone amide resonances. Peak volumes were determined using a newly developed method based on a projection technique [38]. See Supplementary Fig. S2 for a quick summary of the projected volume (Proj-Vol) method, Supplementary Fig. S3 for HSQC1 ^15^N projections, and Supplementary Fig. S4 for EXSY^15^N projections with a mixing time of 0.4 s.

The time-zero HSQC (HSQC0) method was used to suppress the measurement bias originating from unequal coherence transfer efficiencies between the two states in the HSQC pulse sequence (Supplementary Fig. S5) [31,32,34]. Residue-specific equilibrium constants *K*_←*i*_ were determined as the ratio of two cross-peak volumes, *V*_U_(*i*) and *V*_N_(*i*), of the same residue in the HSQC*i* (*i*=1, 2, 3) spectra, and then, unbiased log *K*_←_ values were obtained by extrapolating the log *K*_←*i*_ values (Supplementary Figs. S6 and S7).

The relaxation bias in the EXSY spectra was removed by the Π analysis method [39]. An EXSY spectrum contains two auto peaks and two exchange peaks per residue (Supplementary Fig. S1D). The composite peak ratio Π is defined by a combination of the four peak volumes: two auto peaks, *V*_UU_ and *V*_NN_, and two exchange cross peaks, *V*_UN_ and *V*_NU_, as a function of the mixing time τ.

(2) $$\Pi \left(\tau \right)=\frac{V_{UN}\left(\tau \right)\;V_{NU}\left(\tau \right)}{V_{UU}\left(\tau \right){\;V}_{NN}\left(\tau \right)-V_{UN}\left(\tau \right)\;V_{NU}\left(\tau \right)}$$

The product of the two rate constants *k*_f←_*k*_u←_ of each residue was obtained from the slope of the regression line, using the equation $\sqrt{\Pi }=\sqrt{k_{f\leftarrow }k_{u\leftarrow }}\;\tau$ (Supplementary Figs. S8 and S9). Note that the slope $\sqrt{k_{f\leftarrow }k_{u\leftarrow }}\;$can be determined with high accuracy because there is only one fitting parameter. Although EXSY spectra were measured at multiple mixing times to confirm the linearity of the Π plot, only the 0.4 s mixing time data were used to determine the slopes for the wild-type and V46A proteins (Supplementary Fig. S8A and S8C). For L33A, the data points at mixing times of 0.1, 0.15, 0.2, 0.3, and 0.6 s were included in the regression analysis in addition to the 0.4 s mixing time data, to reduce fitting uncertainty (Supplementary Fig. S8B). See the supplementary material 2 (a zipped Excel file) for details.

Finally, by combining $\sqrt{k_{f\leftarrow }k_{u\leftarrow }}\;$from the EXSY experiment with *K*_←_ (=*k*_f←_/*k*_u←_) from the HSQC0 experiment, residue-specific *k*_f←_ and *k*_u←_ were calculated. Detailed procedures for the analyses of the HSQC*i* and EXSY data were described in the previous study [34].

## Results and discussion

### NMR analysis of the two-state exchange of the spectrin SH3 domain in acidic solutions

We selected the spectrin SH3 domain as a model protein and used all available amide ^1^H nuclei in acidic solutions. The number of ^1^H nuclei used as local probes varied depending on the proteins. The V46A mutant protein had the fewest overlapping cross-peaks and the most residues analyzed, whereas the L33A mutant protein had the most overlapping cross-peaks and the fewest residues analyzed. The wild-type protein was intermediate between the two mutants. We recorded ^1^H-^15^N time-zero HSQC (HSQC0) NMR spectra with different numbers of the HSQC units in the pulse program and ^1^H-^15^N EXSY NMR spectra with different mixing times (Supplementary Fig. S1). Then, we determined the cross-peak volumes using the method based on a projection technique (Supplementary Fig. S2). The projection-based method we developed offers several advantages over conventional cross-peak shape fitting in 2D NMR spectra [38]. First, the projection-based method simplifies the determination of cross-peak *volumes* in 2D spectra to the determination of peak *areas* in 1D projections, resulting in fewer fitting parameters (Supplementary Figs. S3 and S4). Second, adding slices along the ^1^H axis to construct 1D ^15^N projections allows for the elimination of the need to consider ^1^H homonuclear *J*-coupling effects in 2D HSQC*i* and EXSY NMR spectra (Supplementary Fig. S2A and S2B). The singlet peak shapes without *^3^J*_HNHα_ splitting simplifies the spectral analysis. Third, when two cross peaks overlap diagonally in the 2D NMR spectra, narrowing the size of the projection box reduces the intensity of the non-target peak in the 1D projection, leading to a more accurate determination of the projection area of the peak of interest (Supplementary Fig. S2C).

The residue-specific unbiased equilibrium constants log *K*_←_ were obtained from the HSQC0 datasets using the log *K*_←_ vs. HSQC*i* plots (Supplementary Figs. S5, S6, and S7), and the products of the two residue-specific rate constants *k*_f←_*k*_u←_ were obtained from the EXSY datasets using the EXSY-Π plot (Supplementary Figs. S8 and S9). Supplementary Table S1 summarizes log *K*_←_ as well as log *k*_f←_ and log *k*_u←_, calculated using the relationship *K*_←_=*k*_f←_/*k*_u←_.

### Residue-based LFER validation using data from a single spectrin SH3 protein

**Figure 3:**
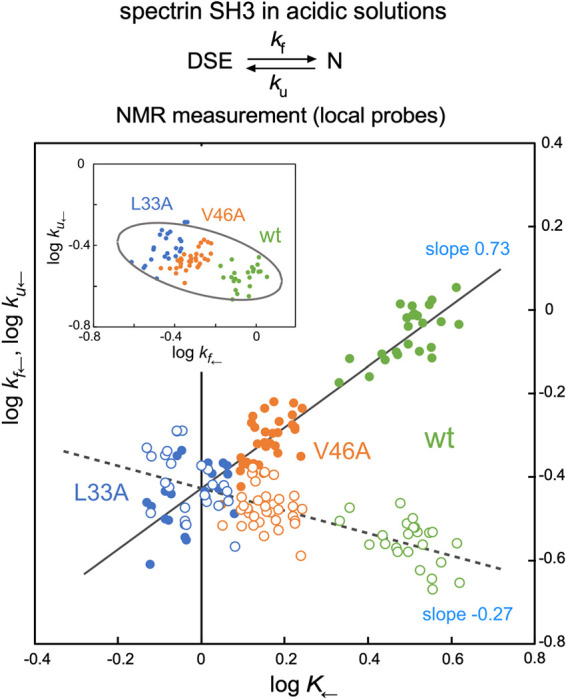
Correlation plots between log *K*_←_, log *k*_f←_, and log *k*_u←_ for the validation of residue-based LFER. Data points are shown in green for the wild-type spectrin SH3 protein, blue for the L33A mutant, and orange for the V46A mutant. Filled data points represent log *k*_f←_, and open data points represent log *k*_u←_. The inset shows the log *k*_u←_ vs. log *k*_f←_ plot with the 95% confidence ellipse.

The residue-specific log *K*_←_ and log *k*_←_ values for the wild-type spectrin SH3 protein were used to generate a log *k*_f←_ vs. log *K*_←_ plot (filled green circles, Fig. 3) and a log *k*_u←_ vs. log *K*_←_ plot (open green circles, Fig. 3). These wild-type data points appear to have a weak linear relationship; however, this is only an apparent weak linear relationship due to the equation log *K*=log *k*_f_–log *k*_u_. Proving the rbLFER relationship requires a negative correlation of data points (or a flat vertical or horizontal distribution of data points) in the third plot, log *k*_u←_ vs. log *k*_f←_ plot (green, inset of Fig. 3) [6]. Unfortunately, this is not the case, so residue-based LFER does not apply for the wild-type spectrin SH3 protein alone. We prepared two single amino acid variants of the spectrin SH3 protein, L33A and V46A, and performed the same NMR experiments and analyses. Just like the wild-type protein, the analysis of either the L33A or V46A mutant alone did not reveal the expected residue-based LFER relationship (blue and orange circles, Fig. 3). The reason for the failed validation was that the variance of residue-specific data points for each protein was insufficient compared to the measurement error.

### Residue-based LFER validation using data from three spectrin SH3 proteins

Interestingly, the data points for the wild-type spectrin SH3 protein are well aligned along a straight line with those for the spectrin SH3 proteins containing the L33A and V46A mutations (Fig. 3). The regression line neatly connects the three clusters of data points (filled circles) in the log *k*_f←_ vs. log *K*_←_ plot, and the other regression line also connects the three clusters of data points (open circles) in the log *k*_u←_ vs. log *K*_←_ plot. The same plot with standard error bars is shown in supplementary Fig. S10 to indicate the precision of the data point locations.

**Figure 4:**
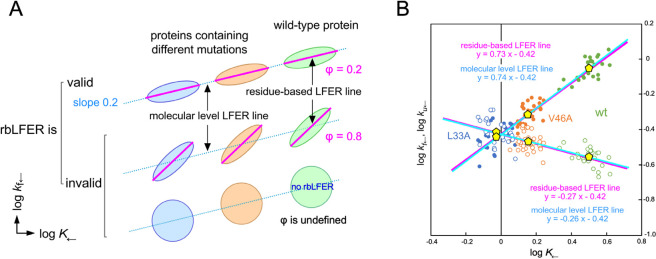
Validation of residue-based LFER. (A) Decision branching for validating residue-based LFER. For rbLFER to be valid, the residue-based LFER lines (magenta lines) within each data point cluster must align parallel to the molecular-level LFER line (cyan lines) passing through the three data point clusters. It is invalid if the slope of the residue-based LFER lines (magenta lines) differs from the slope of the molecular-level LFER line (cyan line) or if there is no correlation in each cluster of data points. (B) Two types of the LFER lines overlap in the case of the spectrin SH3 domain in acidic solution. The residue-based LFER line is the magenta regression line through all data points, whereas the molecular-level LFER line is the cyan regression line through the three centroids (yellow pentagons) of each data point cluster.

Figure 4A summarizes the criteria for data point arrangement. For rbLFER to be valid, the slope of the residue-based LFER line (magenta line) within a data point cluster of the wild-type or mutant proteins must match the slope of the molecular-level LFER line passing through multiple data point clusters of the wild-type and mutant proteins. Ideally, the residue-based LFER lines overlap with the molecular-level LFER line. This feature is not evident in the two mutants but is visible in the wild type. Unfortunately, there are currently two limitations. First, the slopes of the residue-based LFER lines for individual data point clusters (wt, 0.60; L33A, 0.48; V46A, 0.83 in the log *k*_f←_ vs. log *K*_←_ plot) are unreliable due to measurement errors that are not negligible compared with the range of data point dispersion. If rbLFER holds, then the slope of the residue-based LFER line for each cluster should be identical to that of the regression line obtained using all data points. Thus, examining the relationship between log *k*_f←_ and log *K*_←_, reveals the slope of the regression line is 0.73 (magenta, Fig. 4B), which is reasonable alternative to the slope of the residue-based LFER line for each cluster. The second limitation is the lack of experimental equilibrium and rate constants for the two-state exchange of SH3 under acidic conditions measured using a global probe (e.g., Trp fluorescence). As a substitute for such global measurements, we used the average position (centroid) of the local-probe data points. The molecular-level LFER line was estimated by fitting a regression line through the three centroids (yellow pentagons), yielding a slope of 0.74 (cyan, Fig. 4B). This value is essentially identical to the slope of the residue-based LFER line, 0.73. We concluded that these results, combined with the identical intercept value of –0.42 (Fig. 4B), indicate that the residue-based LFER lines overlap with the molecular-level LFER line. Ideally, to overcome the first limitation, it is necessary to develop experimental methods that reduce experimental error and repeat the experiments, and to overcome the second limitation, experiments employing a global probe such as Trp fluorescence should be performed to directly determine the molecular level LFER line. Finally, the three data point clusters corresponding to the wild-type and two mutants combine to show a negative correlation in the log *k*_u←_ vs. log *k*_f←_ plot (inset, Fig. 3). This can be seen by the negative slope of the major axis of the 95% confidence ellipse. The good correlations of the data points in the three log-log plots demonstrate that residue-based LFER is experimentally validated for the two-state exchange equilibrium of the spectrin SH3 domain in acidic solution.

It is possible that the spectrin SH3 protein in acidic solutions has a polarized TSE, but due to the measurement error, rbQFER must be analyzed as rbLFER. Within the limits of measurement accuracy, our tentative conclusion is that the transition state ensemble of the spectrin SH3 domain for the acid-denatured two-state exchange is a diffuse type (Fig. 2A), with the native state accounting for approximately 70% of the TSE, considering that the molecular-level LFER line slope is 0.74 (Fig. 4B).

Local probe experiments using a single protein are ideal to demonstrate rbLFER. However, in reality, the degree of variation in the equilibrium and rate constants is often insufficient compared to the measurement error. The key to success is the use of single amino acid mutations to affect the exchange equilibrium and thereby shift the residue-specific equilibrium and rate constants. As a criterion for selecting amino acid residues to mutate, we examined the protein structure of the spectrin SH3 domain [40]. The hydrophobic side chains of Leu^33^ and Val^46^ are pointing towards the core of the protein. The substitutions to alanine residues were chosen to reduce the side-chain volume of buried hydrophobic residues [14]. The nondestructive deletion mutations, L33A and V46A, reduce the stability of the SH3 protein, but the process of the structural change is expected to remain essentially identical to that of the wild-type protein.

### The residue-based LFER/QFER analysis versus the ϕ-value analysis

The present study extended the applicability of the residue-level analysis based on rbLFER to the structural changes of proteins under acidic conditions. However, the same analysis cannot be applied to protein refolding because currently there are no experimental methods that can accurately measure the residue-level protein refolding from the USE to N states. On the other hand, the ϕ-value analysis, which combines global probe measurements and mutation effects, has not been applied to structural changes other than protein refolding. Therefore, we cannot directly compare the results of residue-level and molecular-level analyses but instead consider the relationship between the two approaches.

The ϕ-value analyses of the spectrin SH3 domain using stopped-flow and tryptophan fluorescence were reported previously [18,41]. To interpret the ϕ values around the side chains of mutated residues, it is common to consider the distribution of ϕ values along amino acid sequences. The amino acid sequence of the spectrin SH3 domain is divided into two regions: ϕ<0.5 in the N-terminal two-thirds and the C-terminal region (res. 8–44+res. 58) and ϕ>0.5 in the C-terminal one-third (res. 46–55) (Fig. 2b in [18] and Fig. 6 in [41]). This implies that the spectrin SH3 protein has a polarized TSE in protein refolding from the USE to N states. Alternatively, the distribution of ϕ values in the transition state of the spectrin SH3 can be estimated by examining the regression lines that pass through the data points in the molecular-level log *k*_f_ vs. log *K* plot (Fig. 7 in [41] shows ln *k* vs. ΔΔG_F-U_/*RT* plot, which is equivalent to the log *k*_f_ vs. log *K* plot). The group average ϕ values are obtained from the slope of the regression lines.

In the following section, we discuss the relationship between the residue-level log *k*_f←_ vs. log *K*_←_ plot used in the rbLFER/QFER analysis and the molecular-level log *k*_f_ vs. log *K* plot used in the ϕ-value analysis. Before proceeding further, we define two terms, “two-state exchange” and “refolding,” to clarify the differences between the terms used in this study. Two-state exchange refers to the folding and unfolding reactions between the unfolded/denatured and native states under conditions where both states are equally stable. A typical example is the folding and unfolding reactions of some SH3 domains under acidic pH conditions. On the other hand, refolding refers to the folding reaction of a protein molecule from a completely unfolded state by removing a denaturant under conditions where the native state is much more stable.

### Relationship between the residue-level plot and the molecular-level plot

**Figure 5:**
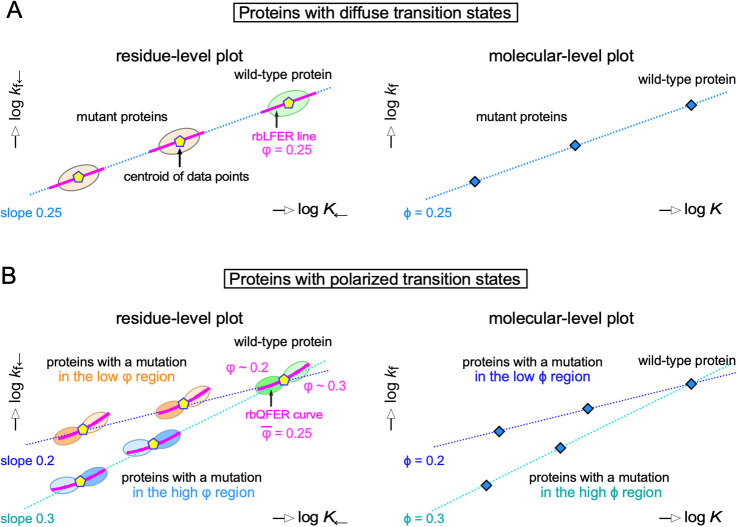
Comparison between residue-level and molecular-level log *k* vs. log *K* plots. (A) Proteins with diffuse TSEs. (*Left*) In the residue-level log *k*_f←_ vs. log *K*_←_ plot constructed from local probe measurements, the tilted ellipse indicates a data point cluster of uniform φ values. The magenta line shows the regression line of rbLFER in the data point clusters. The centroids (yellow pentagons) of the data point clusters lie on a straight line. (*Right*) In the molecular-level log *k*_f_ vs. log *K* plot constructed from global probe measurements, the data points (blue diamonds) from global probe observations lie on a straight line. (B) Proteins with polarized TSEs. (*Left*) In the residue-level log *k*_f←_ vs. log *K*_←_ plot, the pair of tilted ellipses indicates the two separate regions of different φ values. The magenta curve shows the regression parabolic curve of rbQFER in the data point clusters. The centroids (yellow pentagons) of the data point clusters lie on two straight lines. (*Right*) In the molecular-level log *k*_f_ vs. log *K* plot, the data points (blue diamonds) from global probe observations lie on two straight lines.

For diffuse TSE-type proteins (Fig. 5A), in the absence of measurement error, the residue-specific data points of the wild-type protein fall along a magenta straight line in the log *k*_f←_ vs. log *K*_←_ plot due to the residue-based LFER (left panel). However, in reality, measurement error causes the data points to scatter around the magenta line, forming a cluster of data points (green tilted ellipse). The slope ρ of the residue-based LFER line is the φ value (Fig. 2A). When a single mutation is introduced, the cluster moves diagonally left (*i.e.*, both log *k*_f←_ and log *K*_←_ decrease) along a cyan line (orange tilted ellipses). Importantly, the slope of the cyan line is the same as the φ value. The centroids (yellow pentagons) of the clusters of data points lie on the cyan line. Now consider the molecular-level log *k*_f_ vs. log *K* plot generated from the ϕ-value analysis (right panel, Fig. 5A). The data points (blue diamonds) from global probe observations of the wild-type and mutant proteins lie on a straight line. The slope of the line is the ϕ value by definition.

For polarized TSE-type proteins, the situation is a little complicated (Fig. 5B). When a log *k*_f←_ vs. log *K*_←_ plot is constructed from local probe measurements, the data points are arranged on a parabolic curve (magenta curve), log *k*=A(log *K*)^2^+Blog *K*+C, due to the residue-based QFER, in the absence of measurement error (Fig. 2A). The slope of the tangent, 2Alog *K*+B, at a point on the parabolic curve represents the φ value of a residue close to the point. In reality, measurement error scatters the data points around the curve (green ellipses). Here, for simplicity, we assume that there are only two φ values, in which case the data points are divided into two separate regions: one with low φ values and one with high φ values. When a single mutation is introduced into the low φ region, the entire cluster of data points moves along a line with a slope equal to low φ (orange ellipses), and when another single mutation is introduced into the high φ region, the entire cluster of data points moves along a line with a slope equal to high φ (blue ellipses). Similarly, in the log *k*_f_ vs. log *K* plot generated from the ϕ-value analysis, the data points (blue diamonds) from global probe observations of the wild-type and mutant proteins fall on two separate lines (right panel, Fig. 5B). The slopes of the two lines in the molecular-level plot are the ϕ values of the mutated residues by definition.

An important conclusion can be drawn from Figure 5: The ϕ and φ provide equivalent residue-level information about the extent of structure formation in the transition state ensembles. This makes sense, considering that the centroid of a data point cluster is the average of observations from many local probes in the residue-level plot, and therefore the centroid (yellow pentagons) provides a good approximation of the observations from a single global probe (blue diamonds) in the molecular-level plot. As the relationship between the two types of plots in Figure 5 equally applies to all structural changes in proteins, the equivalence between ϕ and φ also applies to all kinds of protein structural changes from fluctuation to refolding (Fig. 2B).

### Real instances of log *k* vs. log *K* plots of proteins with diffuse and polarized TSEs

**Figure 6:**
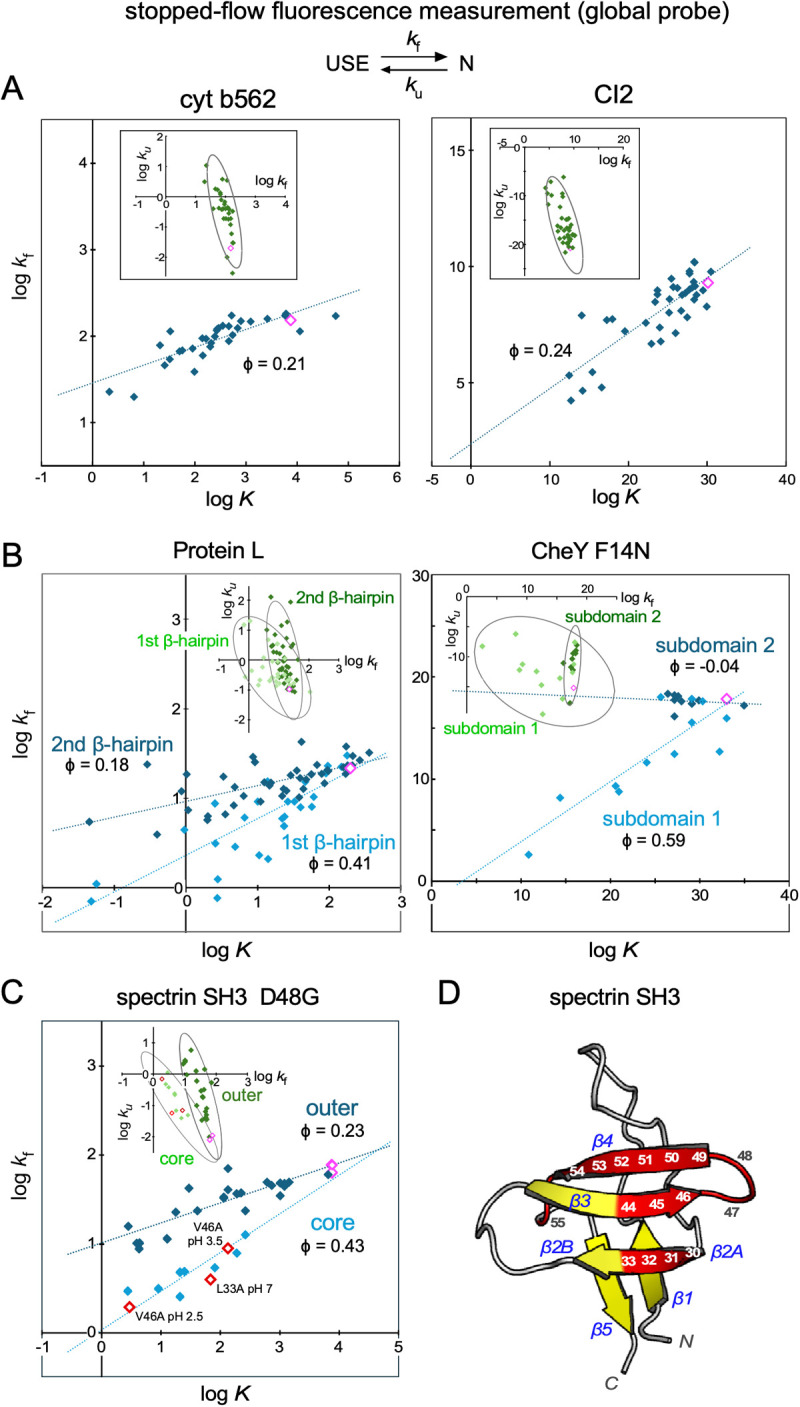
Correlation plots between log *K*, log *k*_f_, and log *k*_u_ of protein refolding from the USE to N states, measured using tryptophan fluorescence as a global probe. The data points (blue and cyan diamonds) represent protein variants with different amino acid mutations. (A) Two exemplary proteins, cytochrome b562 (cyt b562) and chymotrypsin inhibitor 2 (CI2), with diffuse TSEs. The cyt b562 data at pH 5.0, 298 K were obtained from Table 1 in [42]. The CI2 data at pH 6.25, 298 K were from Tables 2 and 3 in [43]. (B) Two exemplary proteins, Protein L and CheY F14N, with polarized TSEs. The Protein L data at pH 7, 295 K were from Table 2 in [44]. The log *K* was calculated from *k*_f_^0.4 M GdmHCl^ and *k*_u_^2 M GdmHCl^. The CheY F14N data at pH 7.0, 298 K were from Table 3 in [45]. (C) Spectrin SH3 is a polarized TSE type. Note that the D48G mutation was introduced to improve the low stability of the wild-type protein in the ϕ-value analyses. Then, a second mutation was introduced by considering the D48G mutant as wild-type SH3, and the effect of the second mutation was investigated [18,41]. The data at pH 2.5, 3.5, and 7.0, 298 K were from Table 1 in [18] and Table 3 in [41]. V46A pH 7.0 was excluded as an outlier. Because Leu^33^ and Val^46^ are both in the core region, the data points corresponding to L33A and V46A are on the same regression line (red open diamonds). In (A), (B), and (C), the magenta open diamond symbols indicate the data points corresponding to the wild-type proteins. (D) The locations of the core residues (res. 30–33 and res. 44–55) are shown on the 3D structure of the spectrin SH3 domain [PDB ID: 1SHG] [40]. The spectrin SH3 domain consists of two three-stranded antiparallel β-sheets. One β-sheet is composed of β-strands β1, β2A, and β5, and the other β-sheet is composed of β2B, β3, and β4, where the β2 strand spans the two β-sheets and is thus divided into β2A and β2B. The cartoon diagram was generated using the PyMOL Molecular Graphics System [46].

Figure 6 shows the log *k*_f_ vs. log *K* plots of some exemplary proteins with diffuse TSEs and polarized TSEs [42–45]. These figures are real instances of the schematic diagram shown in Figure 5. For proteins with diffuse TSEs, the data points corresponding to mutated proteins are aligned along a single line (Fig. 6A), whereas for proteins with polarized TSEs, the data points are aligned along two lines (Fig. 6B). Notably, the two lines intersect at the data point corresponding to the wild-type protein. Figure 6C shows the log *k*_f_ vs. log *K* plot of the spectrin SH3 domain [18,41]. The data points are aligned along two separate lines, indicating that the spectrin SH3 has a polarized TSE in protein refolding under both acidic and physiological conditions. Core and outer residues were defined by assigning residues to one of the two lines in the plot. For example, Leu^33^ and Val^46^ are core residues because the data points for the L33A and V46A mutants lie on the line with a slope of 0.43 (cyan line, Fig. 6C). The assignment of core residues is supported by the fact that residues classified as core residues (res. 30–33+res. 44–55) form a cluster in the 3D structure (Fig. 6D). The average φ value of the core region, 0.43, is larger than that of the outer region, 0.23 (Fig. 6C), indicating that the core region precedes the outer region in structural formation in the transition state. This suggests that the core residues act as folding nuclei in structure formation in protein refolding. It should be noted that the discussion here considers refolding from a fully unfolded state under native conditions, which is different from the two-state exchange under acidic pH conditions already discussed.

### Strategy for demonstrating residue-based LFER/QFER

Local probe experiments using a single protein are ideal to demonstrate rbLFER/QFER. However, in reality, the degree of variation in the equilibrium and rate constants is often insufficient compared to the measurement error. Even in such cases, as shown in this study, the range of the dispersion of equilibrium constants and rate constants can be expanded by introducing amino acid mutations. Experiments to prove residue-based LFER must show that the slope of the residue-based LFER line (magenta line) within a data point cluster of the wild-type or mutant proteins matches the slope of the molecular-level LFER line passing through multiple data point clusters of the wild-type and mutant proteins in log *k*_f←_ vs. log *K*_←_ plots (Fig. 4). In the experiments using diffuse TSE-type proteins, any amino acid residue can be used (Fig. 5A), but in the experiments using polarized TSE-type proteins, mutations must be selected from the same φ-value region (Fig. 5B). To make an appropriate selection, it is useful to refer to the results of ϕ-value analyses (Fig. 6).

### Possibility to prove residue-based LFER/QFER for protein refolding

In this study, we demonstrated that rbLFER holds in the slow exchange between the denatured DSE and native N states of the spectrin SH3 domain in acidic solutions. Nevertheless, demonstrating the effectiveness of rbLFER/QFER in protein refolding under physiological conditions remains a major challenge. The rbLFER/QFER analysis of protein refolding provides experimental evidence that “the consistency principle of protein folding” actually operates in protein refolding.

Protein refolding under native conditions is generally considered to be a unidirectional, irreversible reaction, but it essentially exists in a highly skewed equilibrium. In theory, it should be possible to measure the equilibrium and rate constants for such highly biased reactions, but in practice, no suitable experimental techniques currently exist for measuring accurate residue-level data on protein refolding from the USE to N states. The high demands for measurement accuracy can be relaxed by expanding the variation ranges of equilibrium constants and rate constants by nondestructive deletion mutations. The same goal could be achieved without mutations by altering environmental conditions such as pH, temperature, pressure, ionic strength, type of salt, and type of organic solvent. In conclusion, the idea of combining local probe measurements with the use of multiple proteins containing amino acid mutations is useful (or even essential) for the validation of rbLFER/QFER in various protein-related phenomena, including protein refolding.

## Conflict of interest

The authors note no conflict of interest concerning the research presented here.

## Author contributions

H.S., D.F., and D.K. designed the entire project. H.S. performed experiments and H.S. and D.K. analyzed and visualized data. D.K. wrote the original draft and H.S., D.F., and D.K. reviewed and edited the manuscript.

## Data availability

All data are included in the article and the Supplementary materials. The raw data used to generate Fig. 3, Fig. 4B, Fig. 6, Fig. S6, Fig. S7, Fig. S8, Fig. S9, Fig. S10, and Table S1 are available as Supplementary material 2 (a single zipped Excel file).

## Acknowledgments

This study utilized NMRbox: National Center for Biomolecular NMR Data Processing and Analysis, a Biomedical Technology Research Resource (BTRR) supported by NIH grant P41GM111135 (NIGMS). This work was supported by the Japan Society for the Promotion of Science (JSPS, Japan), KAKENHI Grant Numbers JP21H02448 and JP23K21298, and by the Mitsubishi Foundation (Japan) Research Grants in the Natural Sciences, Grant Number 202110017, to D.K.
