# Supplementary material for: NMR evidence for residue-based LFER relationship in two-state folding-unfolding equilibrium of the spectrin SH3 domain in acidic solutions
Source: Biophys Physicobiol. 2026 Mar 5;23(1):e230011. doi: 10.2142/biophysico.bppb-v23.0011 (PMC13077125; doi:10.2142/biophysico.bppb-v23.0011)
Supplement: Supplementary file 1 — Supplementary material 1 [file 23_e230011_1.pdf]

Supplementary Table S1 Residue-specific equilibrium and rate constants of the two-state exchange of spectrin SH3 proteins in acidic solution

Wild-type[a]	log $K_{\leftarrow}$	SE[e]	$\sqrt{k_{f\leftarrow}k_{u\leftarrow}}$	SE[f]	log $k_{f\leftarrow}$	SE[g]	log $k_{u\leftarrow}$	SE[g]
K6	0.434	0.004	0.478	0.007	-0.104	0.016	-0.537	0.016
V9	0.355	0.008	0.509	0.016	-0.115	0.036	-0.471	0.036
A11	0.518	0.006	0.536	0.020	-0.012	0.040	-0.530	0.040
L12	0.511	0.011	0.546	0.025	-0.008	0.051	-0.519	0.051
Y13	0.553	0.009	0.408	0.011	-0.113	0.031	-0.666	0.031
Y15	0.552	0.010	0.432	0.011	-0.088	0.030	-0.641	0.030
R21	0.331	0.018	0.461	0.015	-0.171	0.041	-0.502	0.041
T24	0.493	0.007	0.547	0.008	-0.016	0.017	-0.509	0.017
T32	0.508	0.008	0.545	0.024	-0.010	0.049	-0.518	0.049
L33	0.468	0.013	0.465	0.014	-0.098	0.037	-0.566	0.037
L34	0.441	0.008	0.459	0.012	-0.118	0.031	-0.558	0.031
scW41[b]	0.547	0.008	0.552	0.017	0.015	0.035	-0.531	0.035
W42	0.619	0.011	0.455	0.011	-0.032	0.031	-0.651	0.031
scW42[b]	0.506	0.012	0.573	0.010	0.011	0.023	-0.495	0.023
K43	0.575	0.010	0.485	0.008	-0.027	0.022	-0.602	0.022
V44	0.613	0.016	0.563	0.016	0.056	0.037	-0.556	0.037
D48	0.403	0.006	0.438	0.020	-0.157	0.048	-0.560	0.048
G51	0.477	0.006	0.601	0.017	0.017	0.031	-0.460	0.031
F52	0.470	0.006	0.456	0.018	-0.106	0.042	-0.576	0.042
V53	0.524	0.009	0.438	0.009	-0.096	0.025	-0.620	0.025
A56	0.528	0.024	0.508	0.010	-0.029	0.032	-0.559	0.032
V58	0.554	0.007	0.560	0.023	0.025	0.044	-0.529	0.044
K59	0.493	0.012	0.469	0.013	-0.080	0.032	-0.577	0.032
L61	0.496	0.006	0.519	0.008	-0.037	0.019	-0.533	0.019

(continued)

L33[c]	log $K_{\leftarrow}$	SE[e]	$\sqrt{k_{f\leftarrow}k_{u\leftarrow}}$	SE[f]	log $k_{f\leftarrow}$	SE[g]	log $k_{u\leftarrow}$	SE[g]
V9	0.052	0.028	0.407	0.036	-0.364	0.102	-0.417	0.102
A11	0.011	0.046	0.355	0.015	-0.445	0.066	-0.456	0.066
Y13	0.015	0.040	0.424	0.007	-0.365	0.037	-0.380	0.037
T24	0.007	0.019	0.366	0.013	-0.433	0.045	-0.440	0.045
G28	-0.001	0.032	0.426	0.006	-0.371	0.029	-0.370	0.029
T32	-0.041	0.022	0.301	0.011	-0.542	0.049	-0.502	0.049
L33	-0.088	0.030	0.410	0.014	-0.431	0.050	-0.344	0.050
N35	-0.079	0.015	0.345	0.016	-0.501	0.054	-0.422	0.054
T37	-0.121	0.010	0.390	0.023	-0.469	0.065	-0.348	0.065
D40	-0.059	0.010	0.482	0.016	-0.347	0.038	-0.287	0.038
scW41[b]	-0.073	0.010	0.396	0.015	-0.439	0.043	-0.365	0.043
W42	0.025	0.040	0.396	0.029	-0.390	0.093	-0.415	0.093
scW42[b]	-0.041	0.011	0.322	0.009	-0.512	0.034	-0.471	0.034
V44	0.079	0.044	0.299	0.023	-0.485	0.100	-0.564	0.100
E45	0.040	0.024	0.358	0.015	-0.426	0.053	-0.466	0.053
D48	-0.047	0.071	0.490	0.016	-0.334	0.069	-0.287	0.069
G51	-0.088	0.016	0.351	0.010	-0.499	0.036	-0.411	0.036
V53	0.063	0.021	0.378	0.020	-0.391	0.064	-0.455	0.064
A56	-0.083	0.016	0.420	0.019	-0.418	0.054	-0.335	0.054
Y57	-0.037	0.016	0.295	0.015	-0.549	0.057	-0.511	0.057
V58	0.061	0.022	0.397	0.010	-0.370	0.037	-0.431	0.037
K59	-0.124	0.019	0.285	0.017	-0.608	0.071	-0.484	0.071
L61	-0.132	0.013	0.404	0.018	-0.459	0.051	-0.327	0.051

V46A[d]	log $K_{\leftarrow}$	SE[e]	$\sqrt{k_{f\leftarrow}k_{u\leftarrow}}$	SE[f]	log $k_{f\leftarrow}$	SE[g]	log $k_{u\leftarrow}$	SE[g]
K6	0.110	0.012	0.383	0.012	-0.362	0.037	-0.472	0.037
E7	0.151	0.012	0.491	0.013	-0.234	0.033	-0.385	0.033
V9	0.149	0.015	0.343	0.020	-0.390	0.066	-0.538	0.066
A11	0.171	0.012	0.419	0.008	-0.293	0.025	-0.463	0.025
L12	0.095	0.008	0.400	0.018	-0.351	0.049	-0.446	0.049
Y13	0.219	0.007	0.415	0.009	-0.273	0.024	-0.491	0.024

D14	0.241	0.014	0.443	0.044	-0.233	0.107	-0.474	0.107
Y15	0.184	0.008	0.369	0.015	-0.341	0.045	-0.524	0.045
E22	0.164	0.011	0.399	0.021	-0.317	0.058	-0.481	0.058
G28	0.113	0.009	0.375	0.012	-0.370	0.038	-0.483	0.038
L31	0.224	0.012	0.407	0.019	-0.278	0.053	-0.502	0.053
T32	0.153	0.008	0.429	0.027	-0.290	0.066	-0.444	0.066
L33	0.184	0.013	0.413	0.014	-0.292	0.042	-0.476	0.042
L34	0.186	0.023	0.440	0.019	-0.264	0.054	-0.450	0.054
N35	0.134	0.004	0.411	0.012	-0.319	0.031	-0.453	0.031
D40	0.173	0.009	0.496	0.005	-0.218	0.016	-0.391	0.016
W41	0.218	0.012	0.439	0.028	-0.249	0.070	-0.467	0.070
scW41[b]	0.155	0.005	0.396	0.016	-0.325	0.044	-0.480	0.044
W42	0.224	0.015	0.402	0.011	-0.284	0.035	-0.508	0.035
scW42[b]	0.095	0.011	0.340	0.012	-0.421	0.041	-0.516	0.041
K43	0.177	0.010	0.388	0.017	-0.323	0.050	-0.500	0.050
V44	0.238	0.007	0.341	0.022	-0.348	0.069	-0.586	0.069
E45	0.120	0.006	0.470	0.016	-0.268	0.038	-0.388	0.038
V46	0.137	0.009	0.366	0.008	-0.368	0.026	-0.506	0.026
D48	0.125	0.005	0.354	0.013	-0.388	0.039	-0.513	0.039
R49	0.122	0.014	0.486	0.012	-0.252	0.031	-0.374	0.031
G51	0.109	0.006	0.382	0.010	-0.363	0.030	-0.472	0.030
V53	0.152	0.008	0.406	0.011	-0.315	0.032	-0.467	0.032
A55	0.049	0.016	0.325	0.017	-0.464	0.060	-0.513	0.060
A56	0.125	0.002	0.372	0.008	-0.366	0.024	-0.492	0.024
V58	0.222	0.029	0.467	0.007	-0.220	0.030	-0.442	0.030
K59	0.093	0.010	0.372	0.013	-0.383	0.041	-0.476	0.041
K60	0.100	0.004	0.383	0.010	-0.367	0.028	-0.466	0.028
L61	0.128	0.008	0.454	0.009	-0.279	0.023	-0.407	0.023

[a]1.0 mM spectrin SH3 protein wild-type at pH 2.4, 298 K. [b]Indole NH group of tryptophan side chain. [c]2.2 mM spectrin SH3 protein L33A at pH 3.6, 298 K. [d]1.8 mM spectrin SH3 protein V46A at pH 2.4, 298 K. [e]SE is the standard error of the regression intercept of the log K vs. HSQCi plots. [f]SE is the standard error of the regression slope in the EXSY-Π plots. [g]SE is the standard error calculated from the SEs of log $K_{\leftarrow}$ and $\sqrt{k_{f\leftarrow}k_{u\leftarrow}}$ using error propagation.

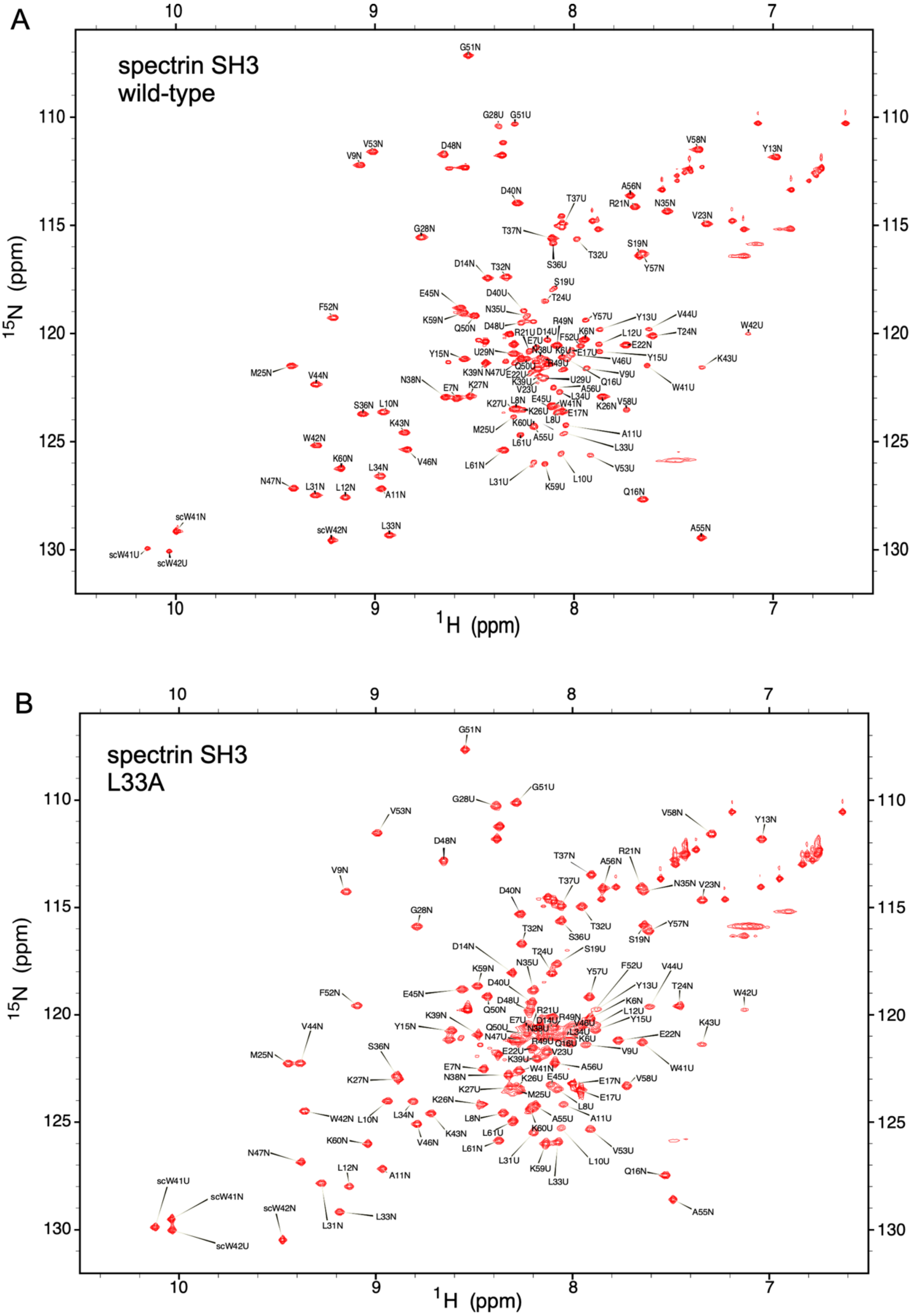
A
spectrin SH3
wild-type
^{15}N (ppm)
^{1}H (ppm)
B
spectrin SH3
L33A
^{15}N (ppm)
^{1}H (ppm)

(continued)

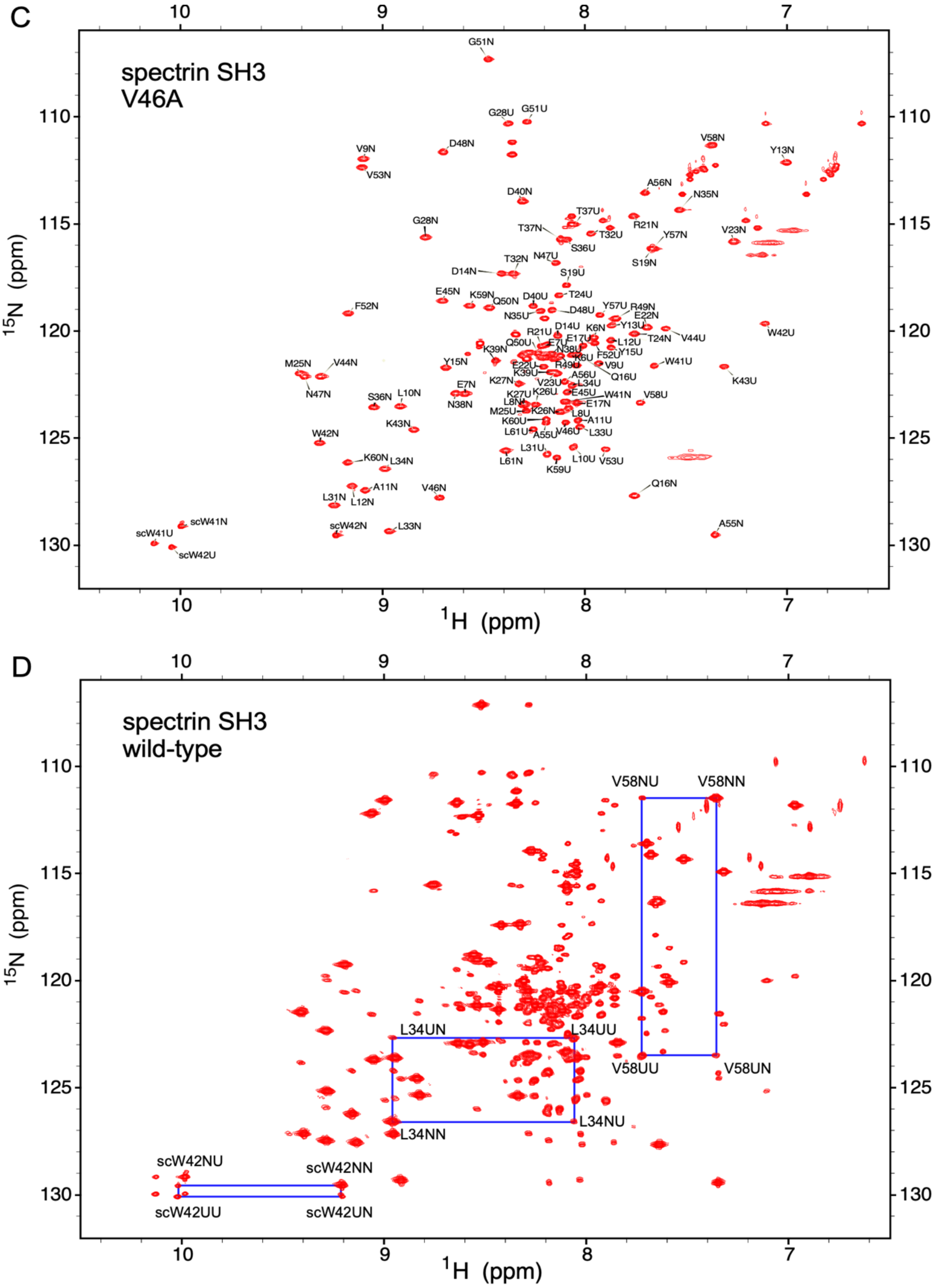

Supplementary Figure S1 ^{1}H-^{15}N HSQC spectra of (A) the wild-type spectrin SH3 protein and its mutants, (B) L33A and (C) V46A, and (D) ^{1}H-^{15}N EXSY spectrum (with a mixing time of 0.4 s) of the wild-type spectrin SH3.

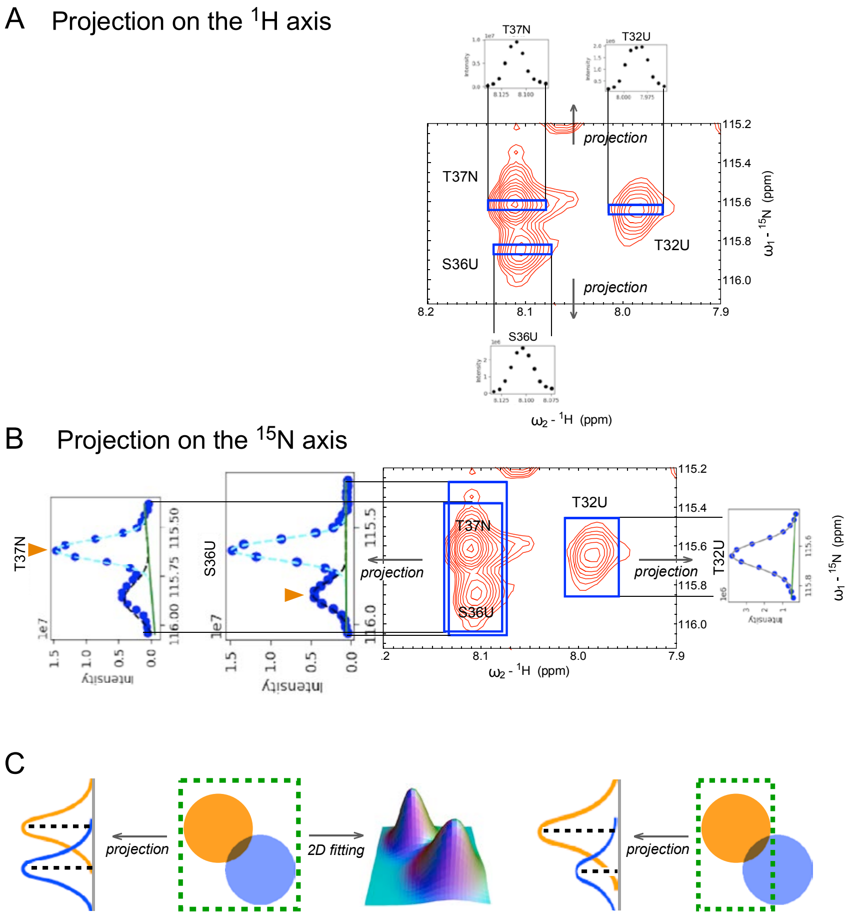

Supplementary Figure S2 Peak volume determination using a projection technique. (A) The initial position of a cross peak in ppm is input from a peak list. In the first step, Δx (number of points in ^{1}H projection) is optimized with reference to the ^{1}H projection spectrum, while Δy (number of points in ^{15}N projection) is fixed at 3. Δx is adjusted to enclose the peak of interest and minimize the baseline on either side. No fitting is performed in this step. (B) In the next step, Δy is optimized with reference to the ^{15}N projection spectrum, while Δx is set to the value determined in the first step. For isolated cross peaks, Δy is adjusted to encompass the peak of interest, and the peak area of the fitted single Gaussian function is calculated. For partially overlapping or closely spaced peaks, Δy is set to include all peaks, and the peak areas of the two or three fitted Gaussian functions are calculated separately. The calculated areas for specified peaks are exported to a text file as peak volumes. (C) When two cross peaks overlap diagonally in a 2D NMR spectrum, conventional 2D cross-peak shape fitting methods require a rectangular region large enough to encompass the two cross peaks (left). Narrowing the size of the projection box reduces the intensity of the non-target peak in the 1D projection, allowing for a more accurate determination of the projected area of the peak of interest (right).

A ^{15}N projection of HSQC1 (spectrin SH3 wild-type)

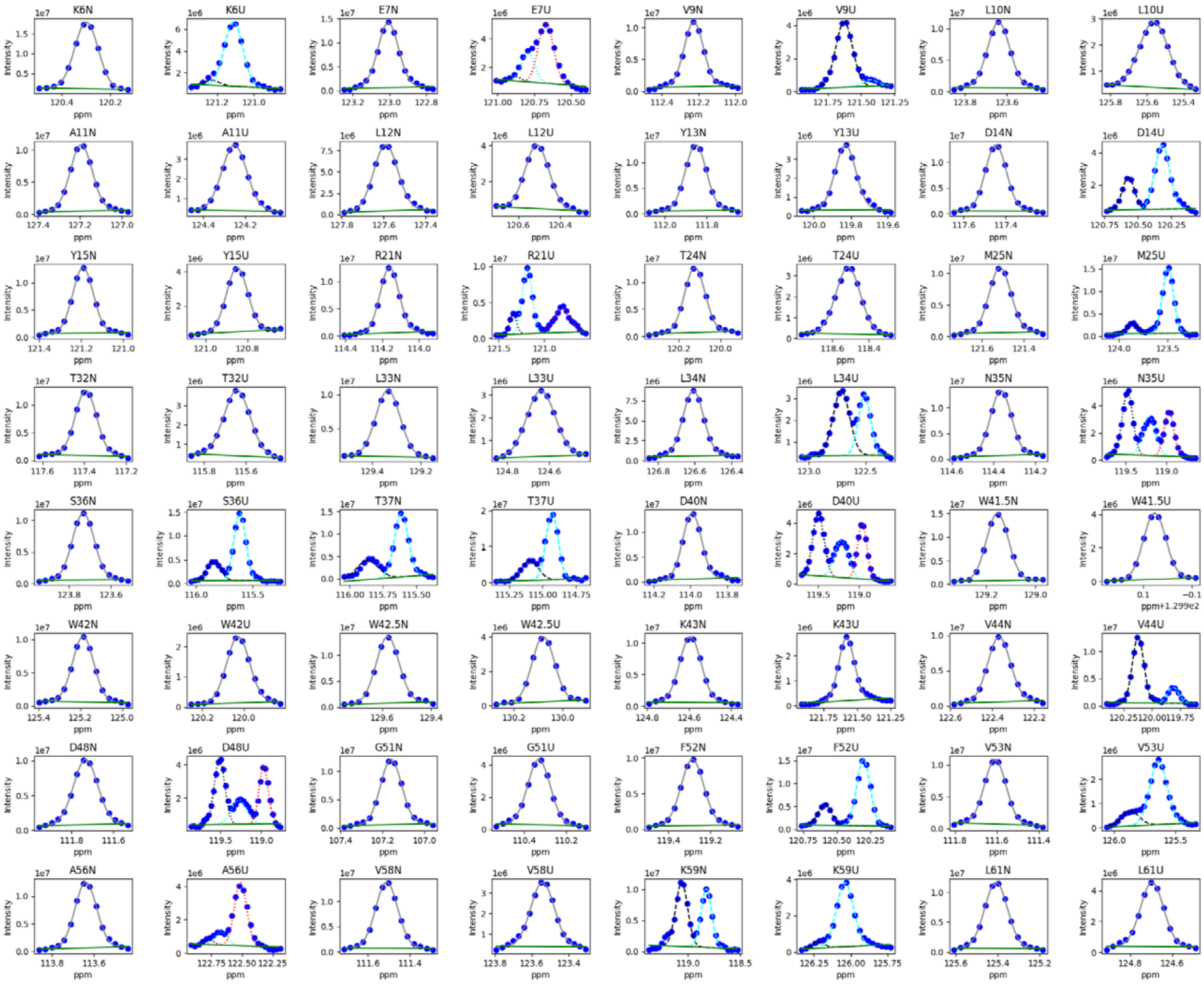

(continued)

B ^{15}N projection of HSQC1 (spectrin SH3 L33A)

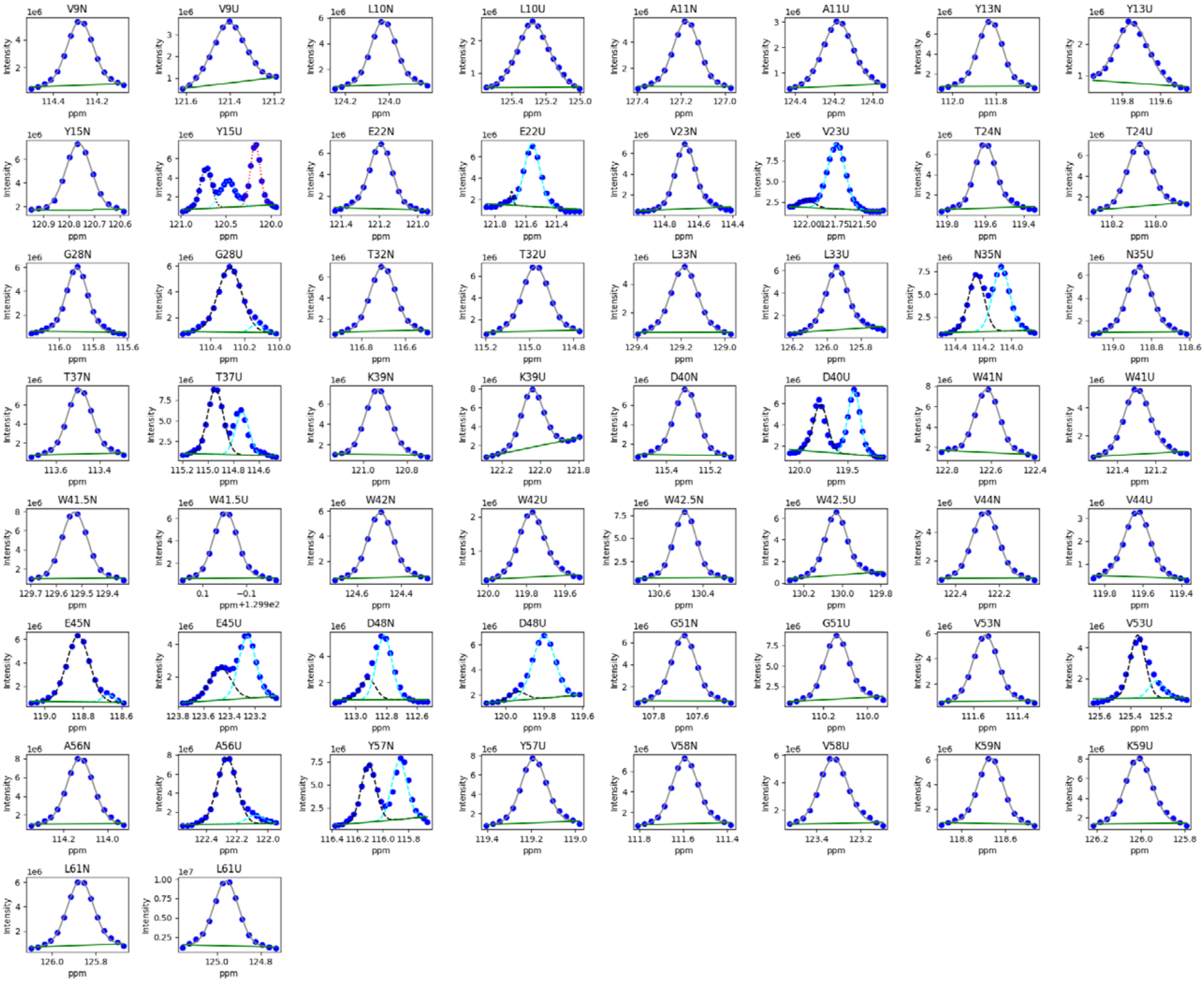

(continued)

C ^{15}N projection of HSQC1 (spectrin SH3 V46A)

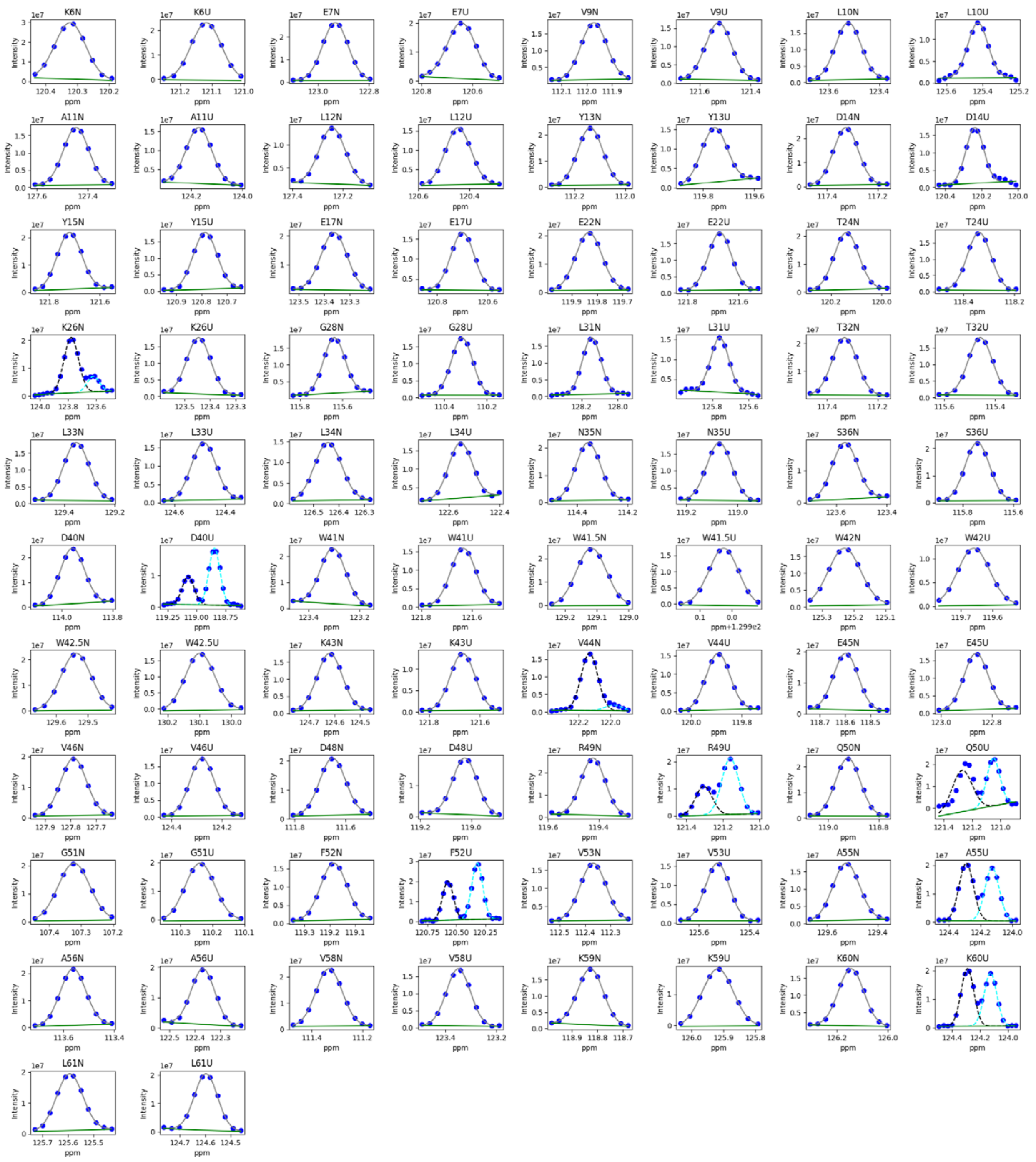

Supplementary Figure S3 Projections of the cross peaks in HSQC1 onto the ^{15}N axis. Experimental NMR data points are shown as blue circles. The curves fitted with a single Gaussian function are shown as solid gray lines, the curves fitted with two Gaussian functions are shown as dashed cyan and dashed black lines, and the curves fitted with three Gaussian functions are shown as dotted cyan, dotted black, and dotted red lines. The solid green lines indicate the linear baseline corrections.

A ^{15}N Projection of EXSY with a mixing time of 0.4 s (spectrin SH3 wild-type)

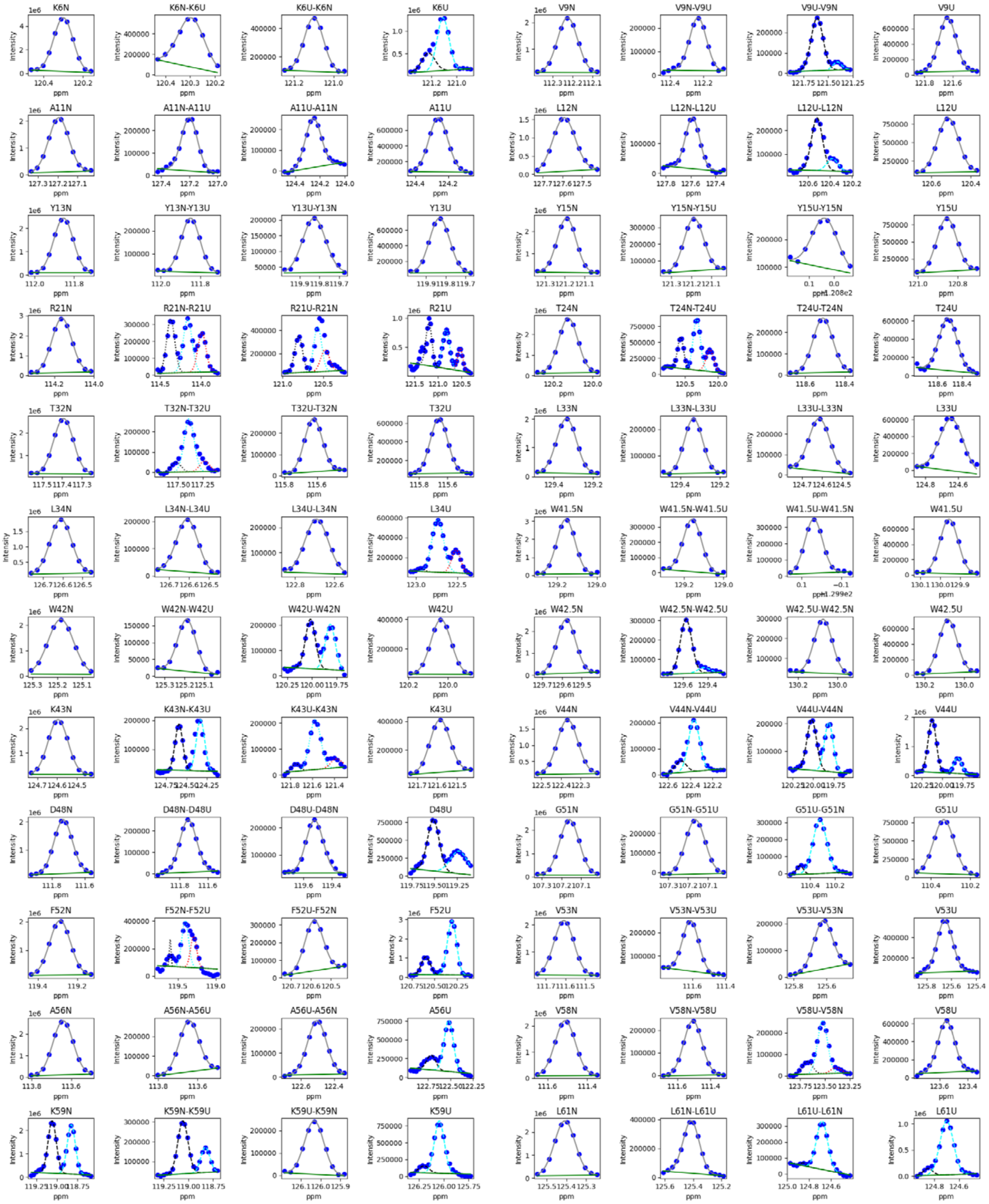

(continued)

B [15]N Projection of EXSY with a mixing time of 0.4 s (spectrin SH3 L33A)

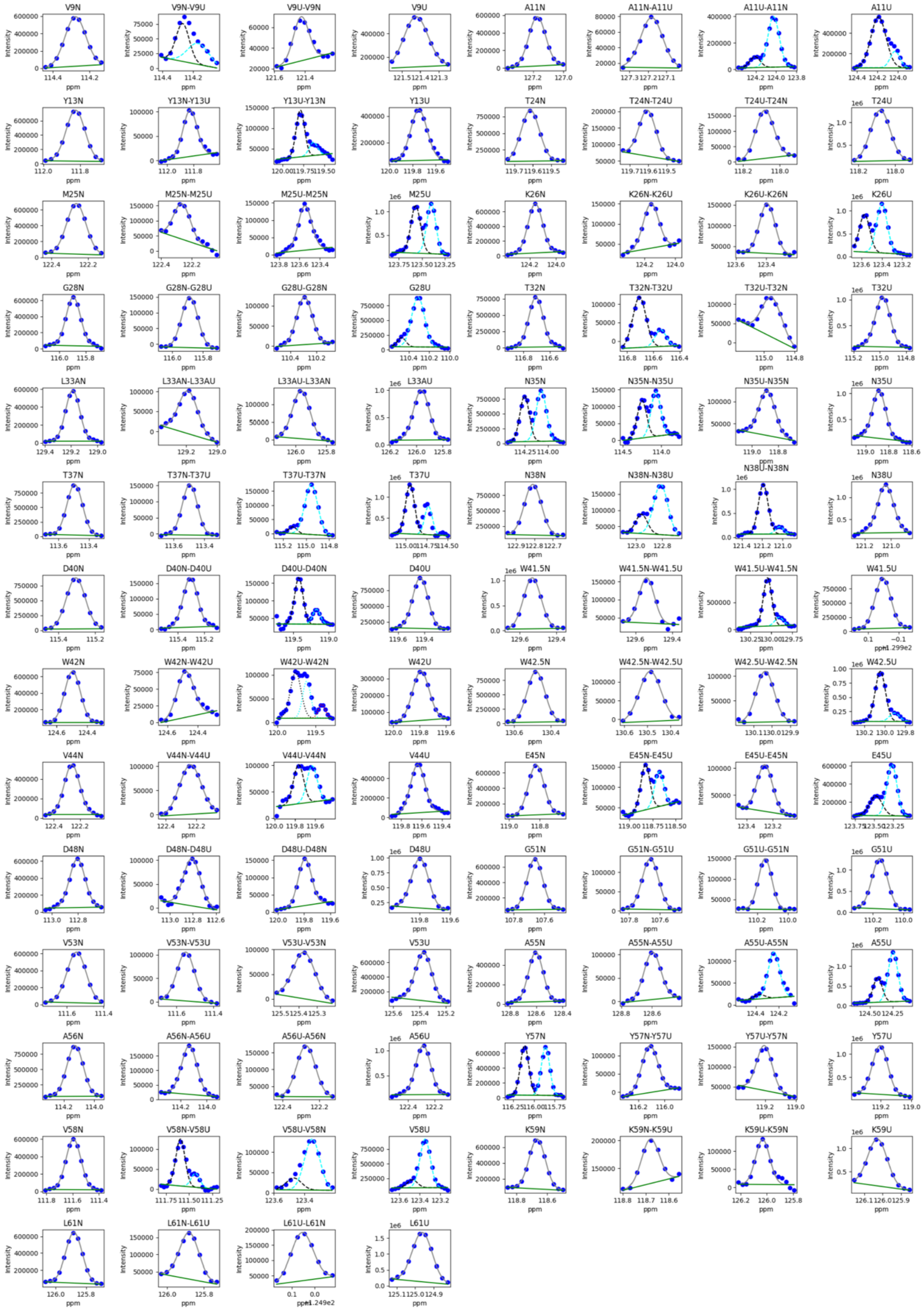

(continued)

C ^{15}N Projection of EXSY with a mixing time of 0.4 s (spectrin SH3 V46A)

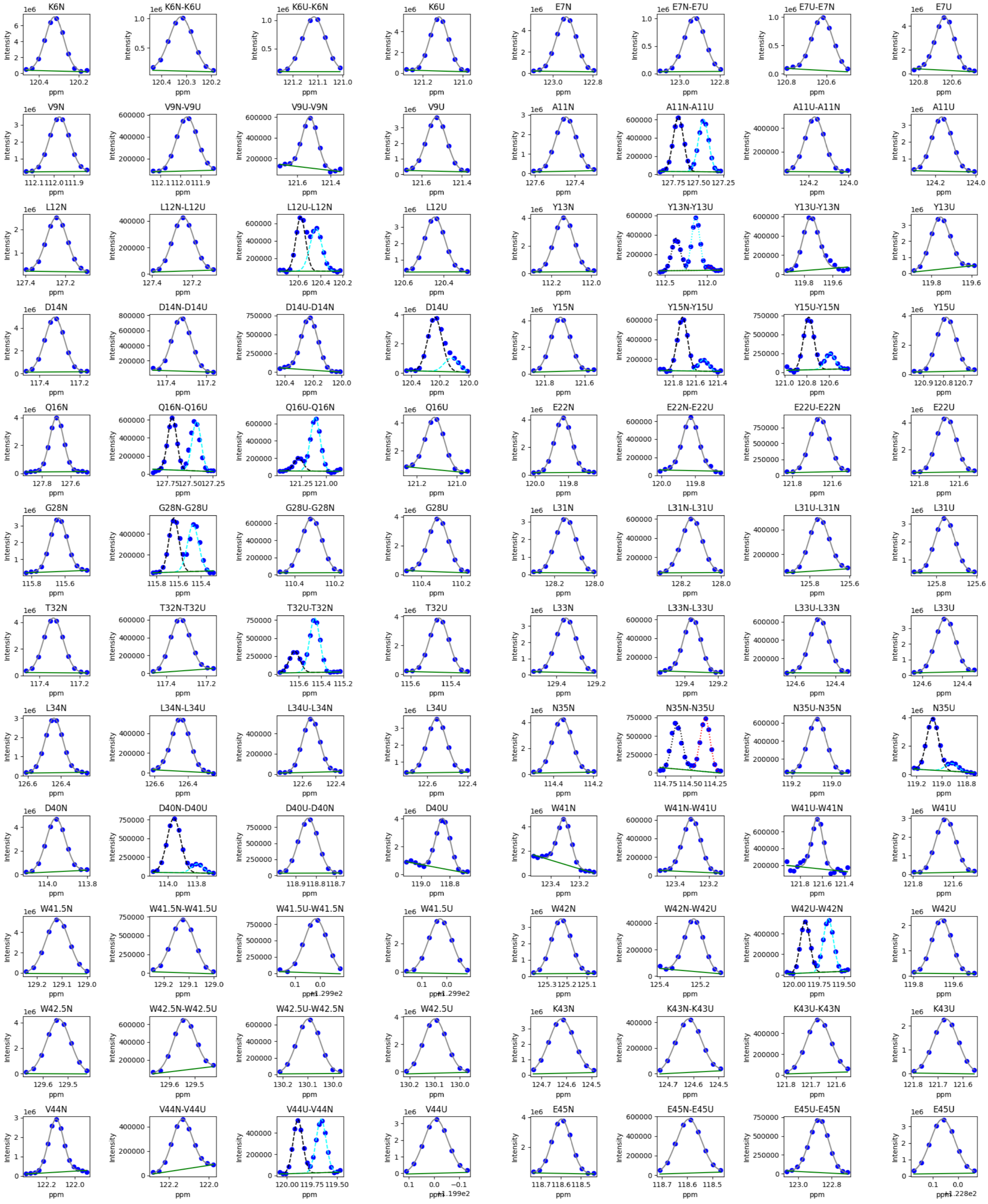

(continued)

C (page 2) ^{15}N Projection of EXSY with a mixing time of 0.4 s (spectrin SH3 V46A)

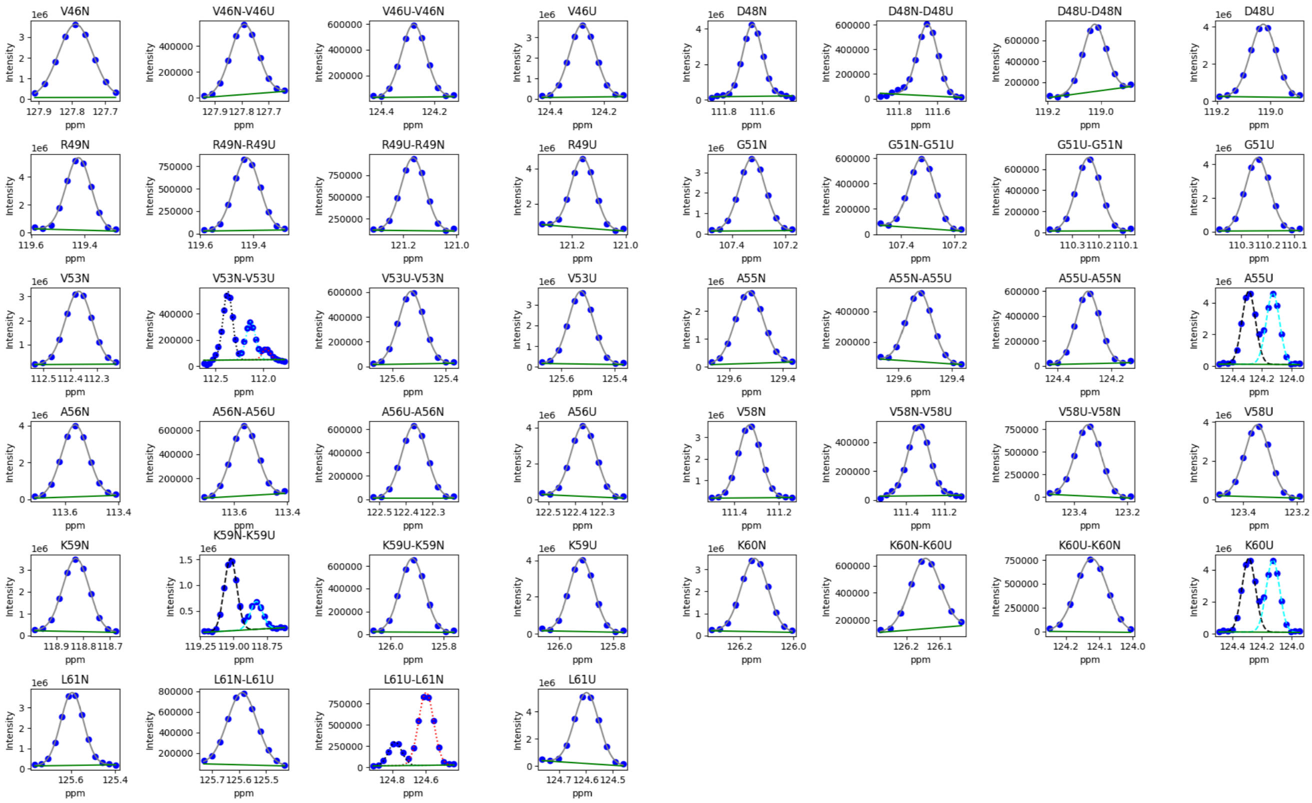

Supplementary Figure S4 Projections of the cross peaks in EXSY with a mixing time of 0.4 s onto the ^{15}N axis. Experimental NMR data points are shown as blue circles. The curves fitted with a single Gaussian function are shown as solid gray lines, the curves fitted with two Gaussian functions are shown as dashed cyan and dashed black lines, and the curves fitted with three Gaussian functions are shown as dotted cyan, dotted black, and dotted red lines. The solid green lines indicate the linear baseline corrections.

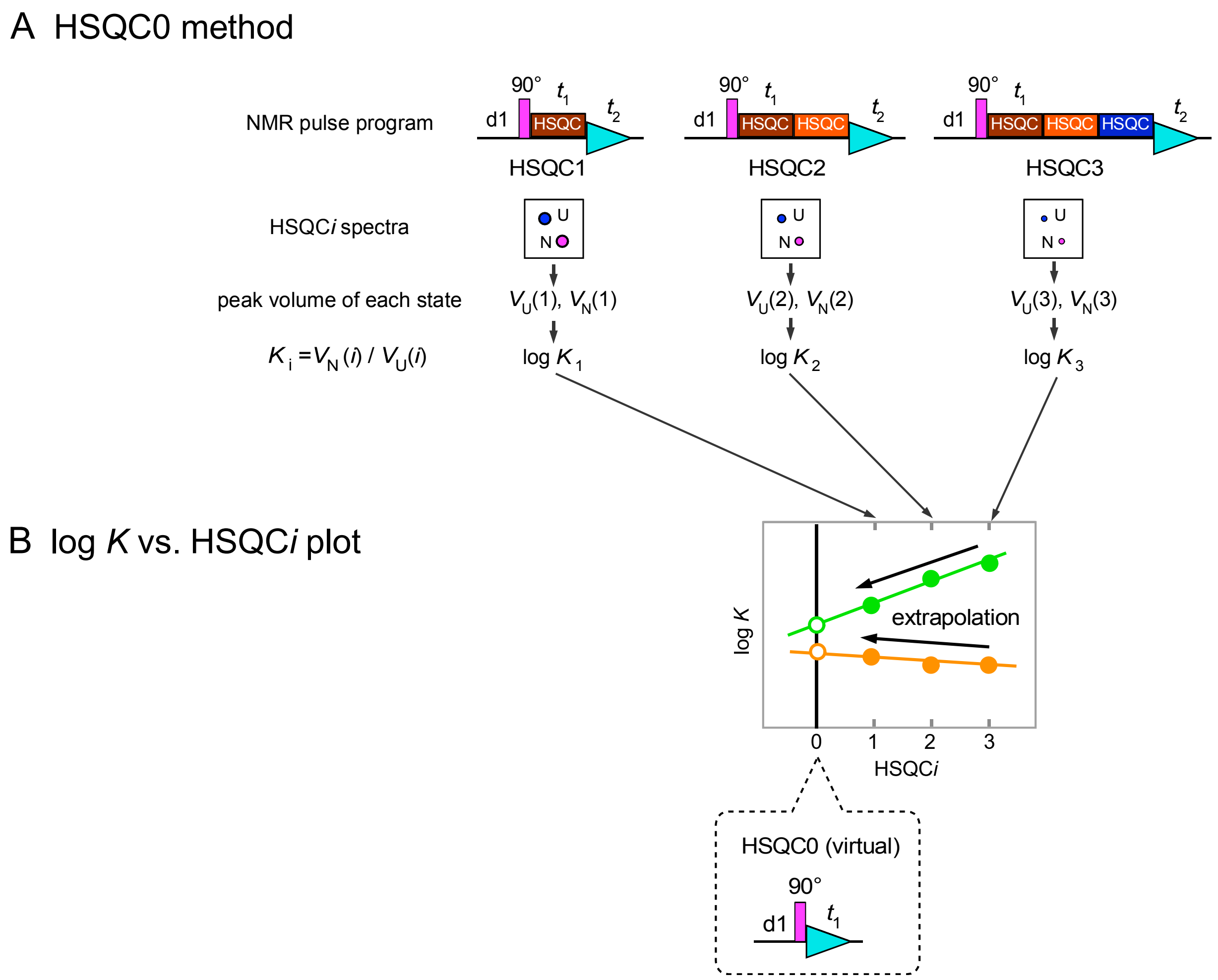

Supplementary Figure S5 Outline of the time-zero HSQC (HSQC0) method. (A) A series of 2D NMR spectra is recorded with an increasing number of basic HSQC blocks. One HSQC0 experiment consists of three pulse sequences containing one HSQC block (HSQC1), two blocks (HSQC2), and three blocks (HSQC3). HSQC1 is identical to the conventional HSQC. In each HSQC*i* spectrum, the peak volumes V_U and V_N are determined for cross peaks corresponding to the two states of the same ^{1}H nuclei, and the ratio of the two cross-peak volumes is calculated as the equilibrium constant K_i. (B) The bias-free equilibrium constant K_0 is obtained through linear extrapolation of the K_i data points. This extrapolation is equivalent to acquiring a 1D NMR spectrum with a single pulse. The measurement error of log K_0 was estimated as the standard error (SE) of the regression intercept. To reduce the standard error, the HSQC0 experiments were repeated to increase the number of log K_i data points.

A log $K_{\leftarrow}$ vs. HSQCi plot (spectrin SH3 wild-type)

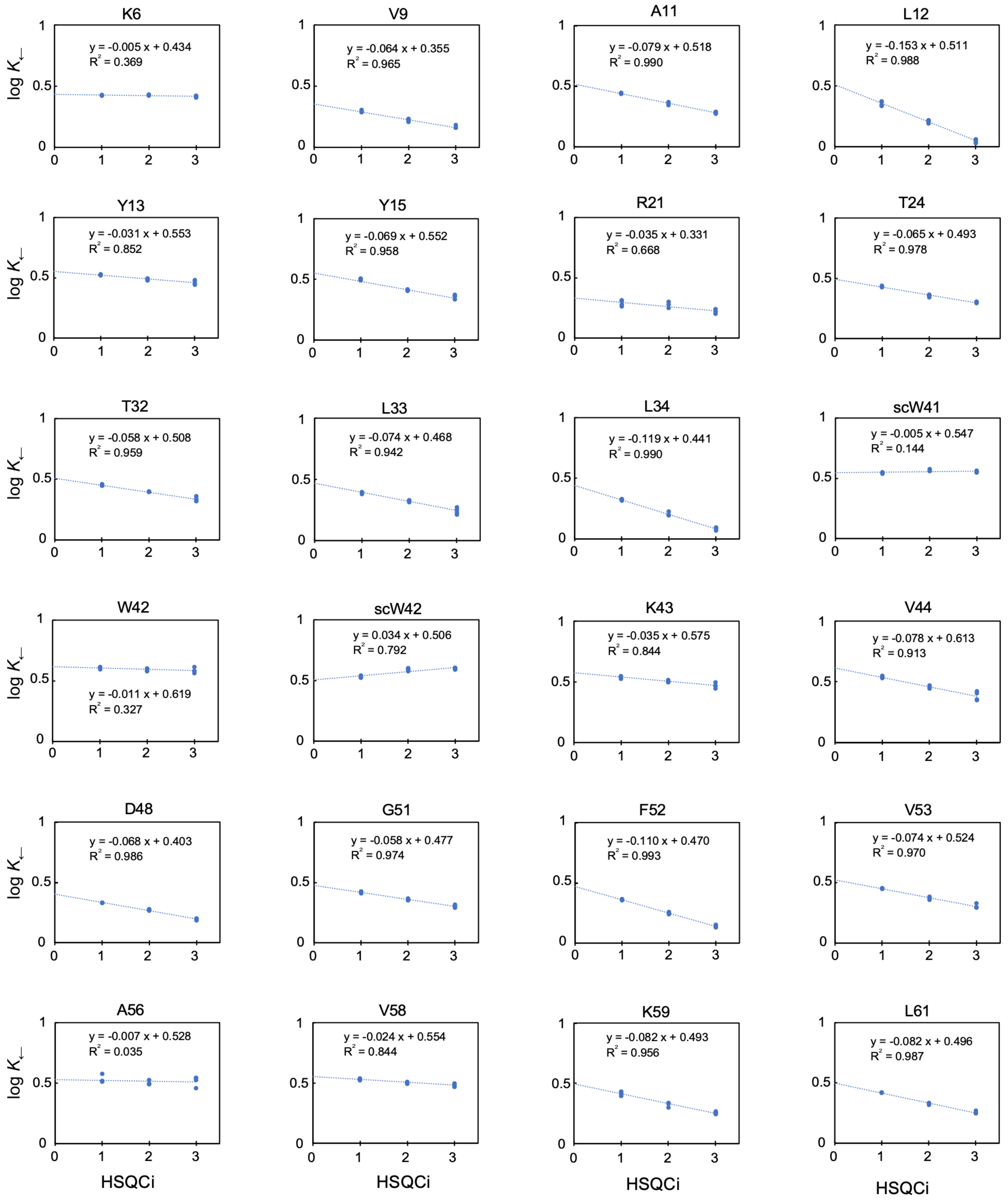

(continued)

B log *K*← vs. HSQC*i* plot (spectrin SH3 L33A)

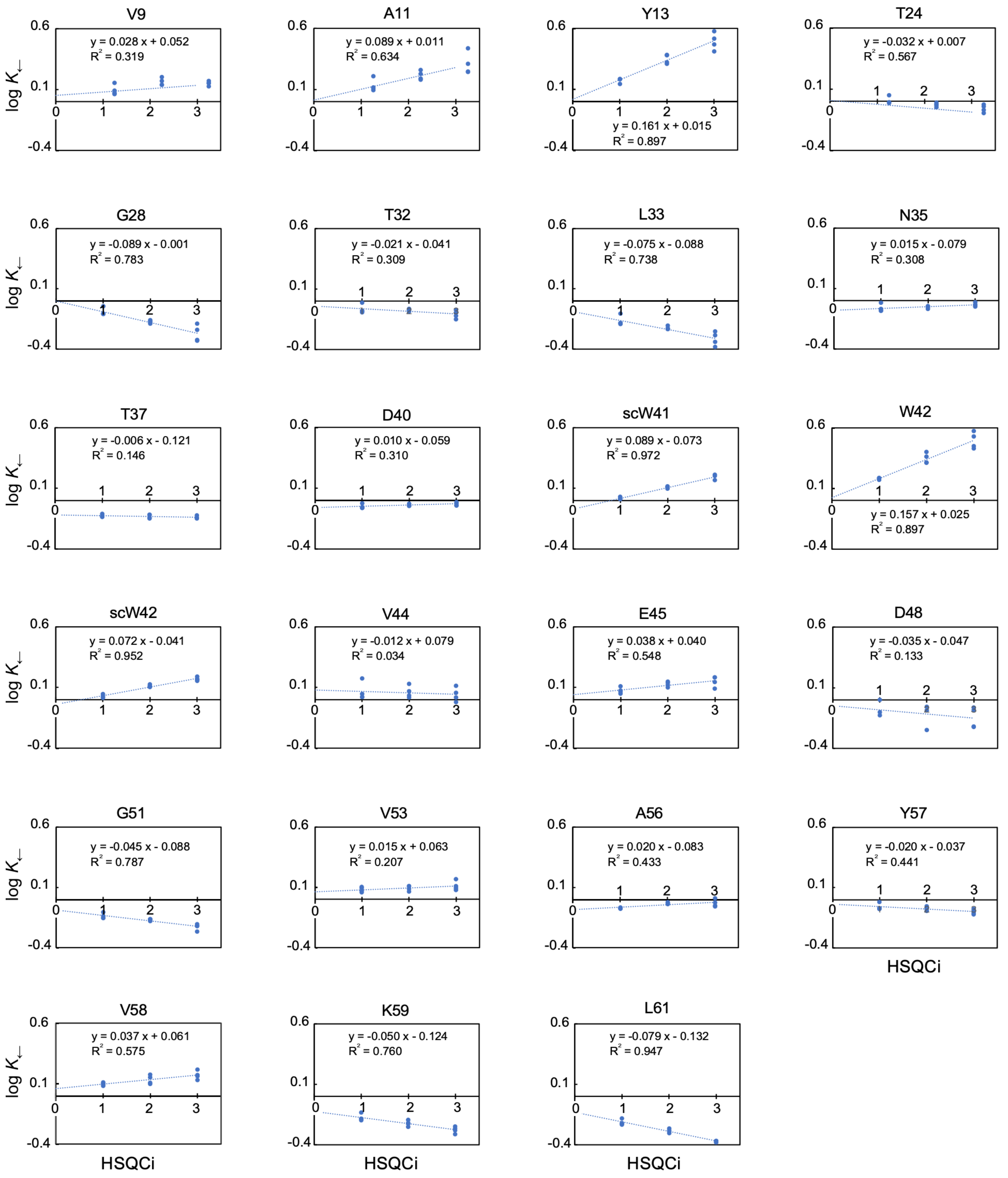

(continued)

C log $K_{\leftarrow}$ vs. HSQC*i* plot (spectrin SH3 V46A)

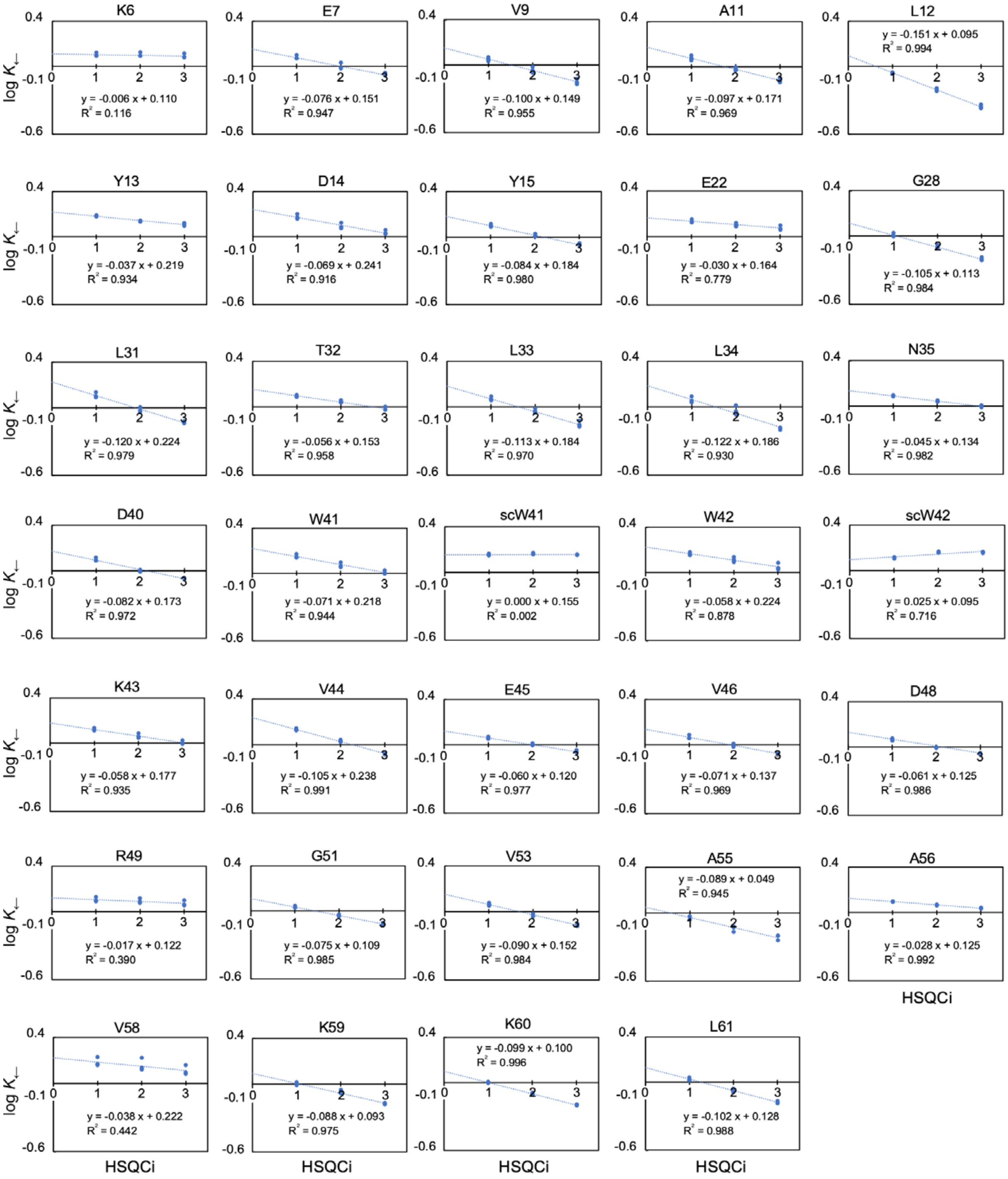

Supplementary Figure S6 Log $K_{\leftarrow}$ vs. HSQC*i* plots. (A) Wild-type spectrin SH3 protein, (B) L33A mutant, and (C) V46A mutant. Each HSQC*i* (i = 1, 2, 3) measurement was repeated four times. Some plots may appear to have fewer points than others, but this is due to small variations in the log $K_{i\leftarrow}$ values or the exclusion of some data points as outliers. See the supplementary Dataset_S1.xlsx for details.

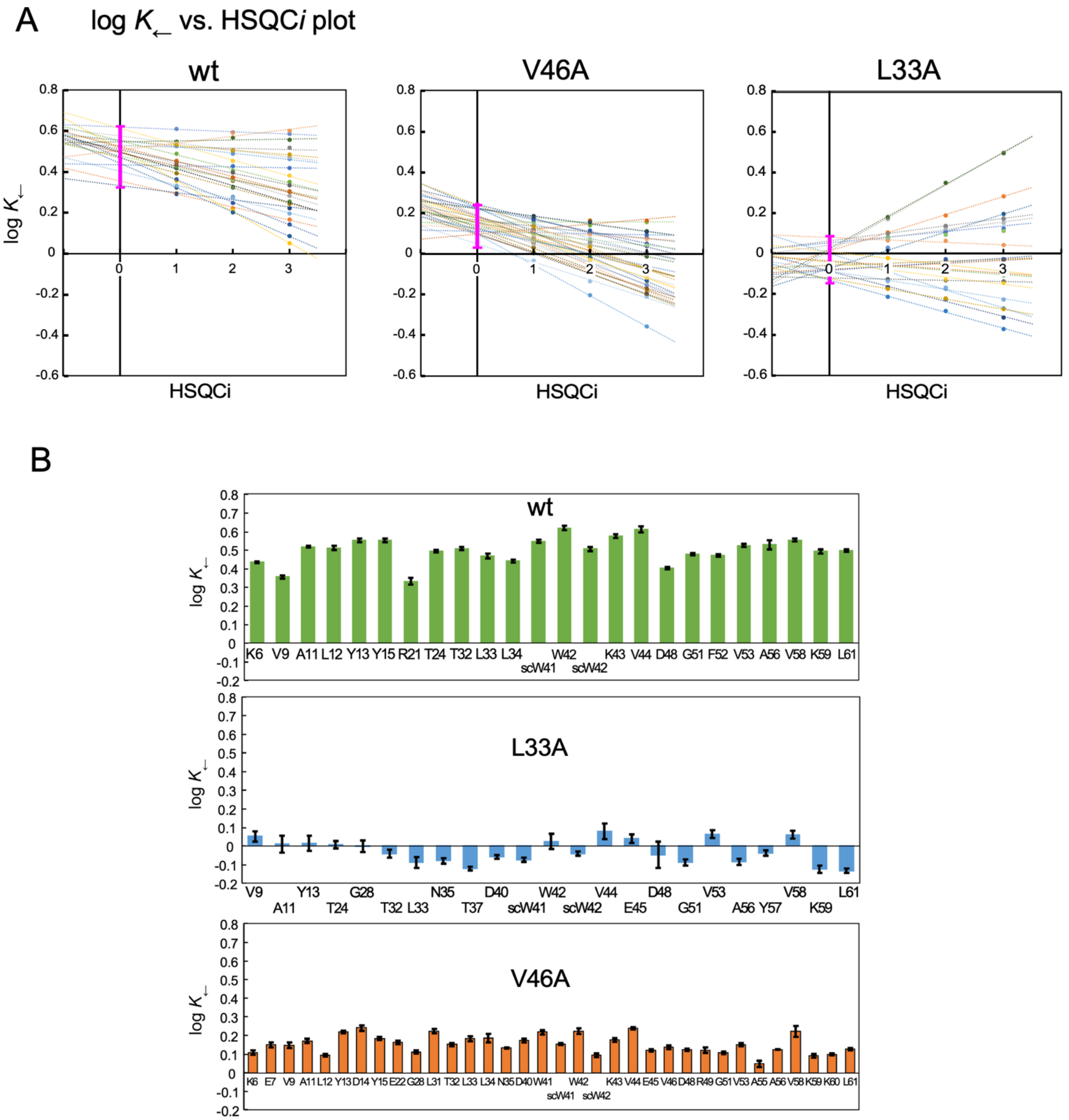

Supplementary Figure S7 Distribution of the equilibrium constants at the residue level. (A) Combined log $K_{\leftarrow}$ vs. HSQC*i* plots. For clarity, instead of showing all individual data points, the average for each HSQC*i* experiment is displayed as a data point. The vertical magenta bars indicate the range of the bias-free log $K_{\leftarrow}$ values. (B) Bar graphs showing the bias-free log $K_{\leftarrow}$ value of each residue. Error bars represent the standard error (SE) of the regression intercept.

A EXSY-Π plot (spectrin SH3 wild-type)

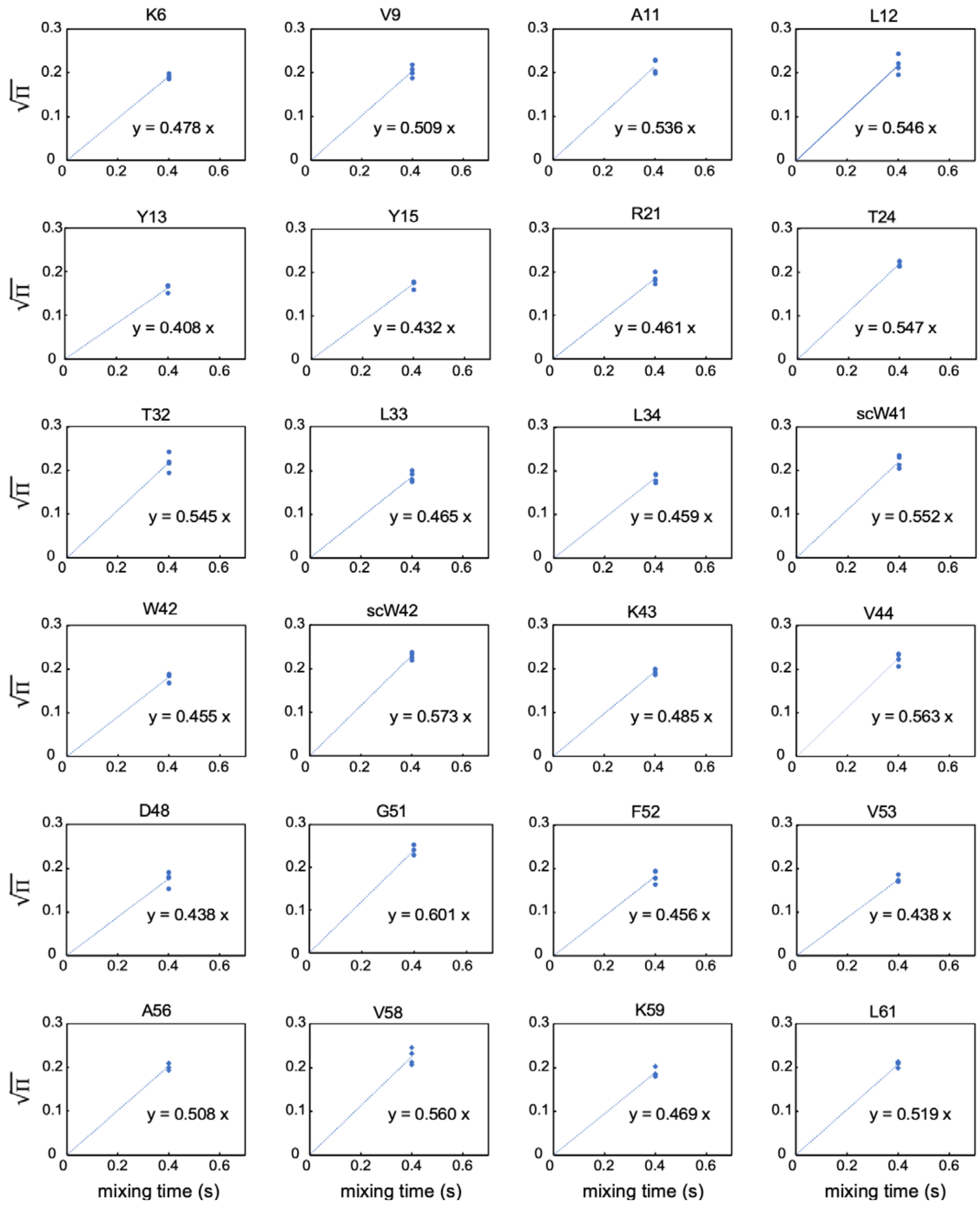

(continued)

B EXSY-Π plot (spectrin SH3 L33A)

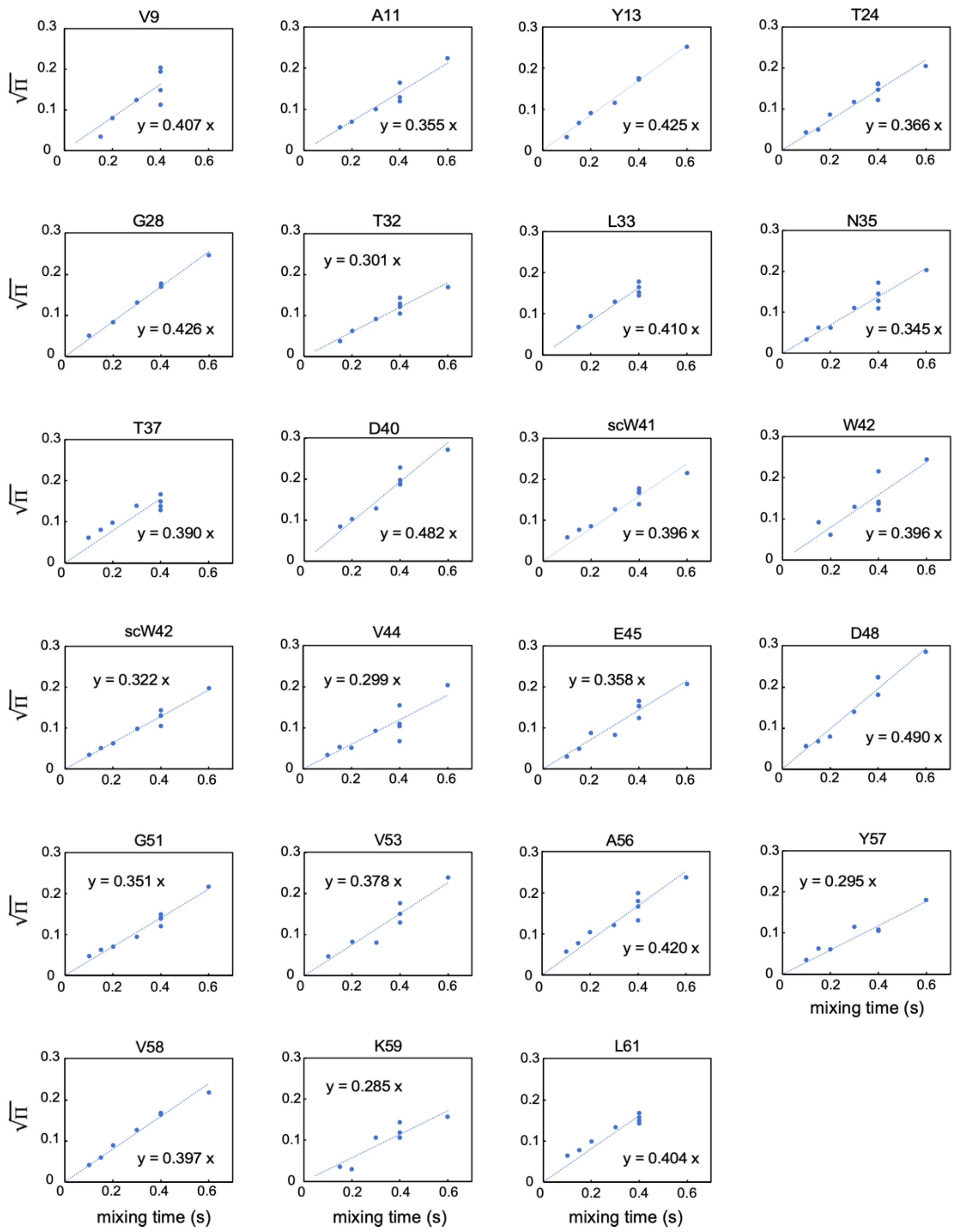

(continued)

C EXSY-Π plot (spectrin SH3 V46A)

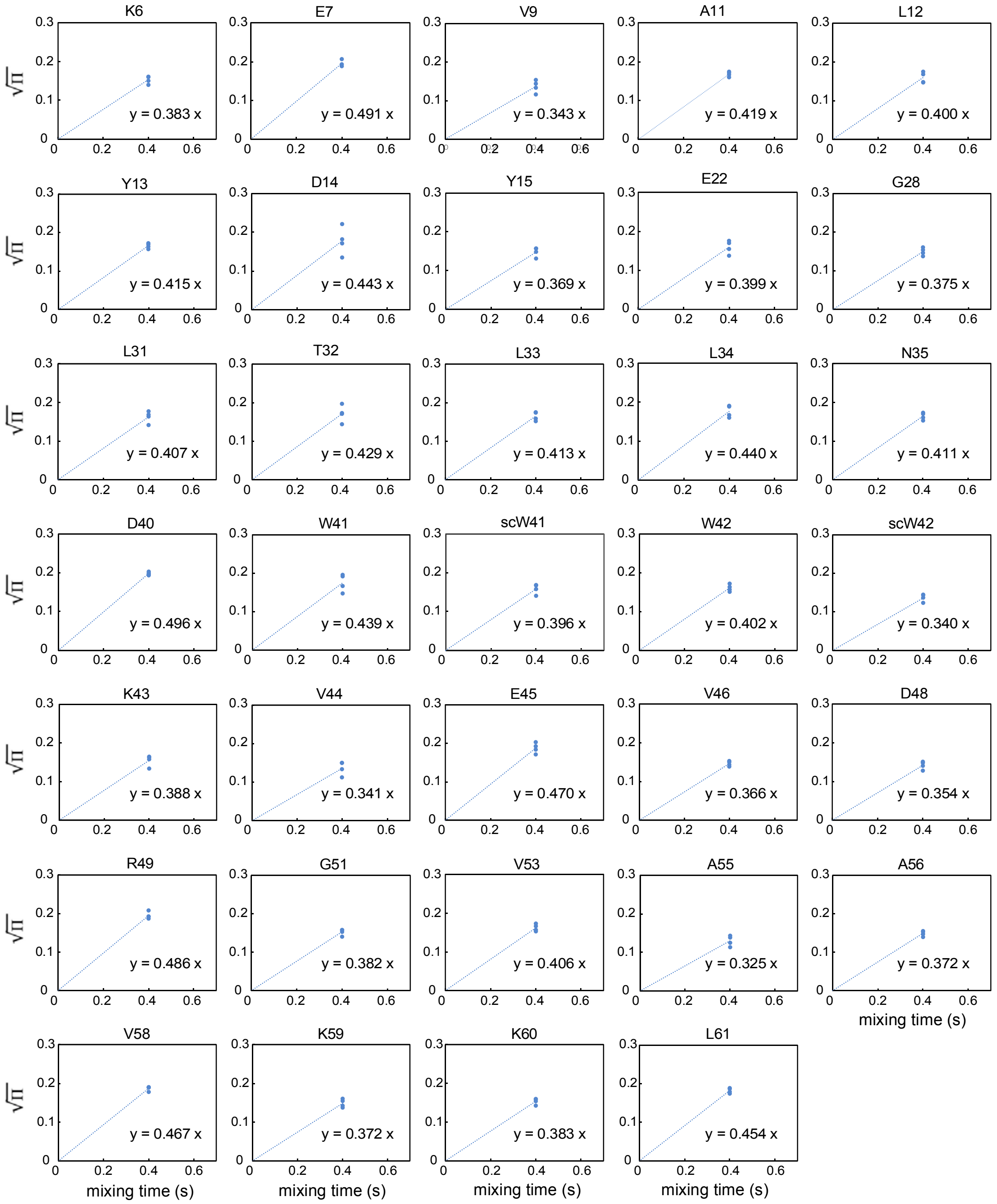

Supplementary Figure S8 EXSY-Π plots. (A) Wild-type spectrin SH3 protein, (B) L33A mutant, and (C) V46A mutant. The EXSY measurement with a mixing time of 0.4 s was repeated four times. Other mixing time data points were used to confirm linearity but were not included in the analysis of wild-type and V46A. Some plots may appear to have fewer points than others, but this is due to small variations in the $\sqrt{\Pi}$ values or the exclusion of some data points as outliers. See the supplementary Dataset_S1.xlsx for details.

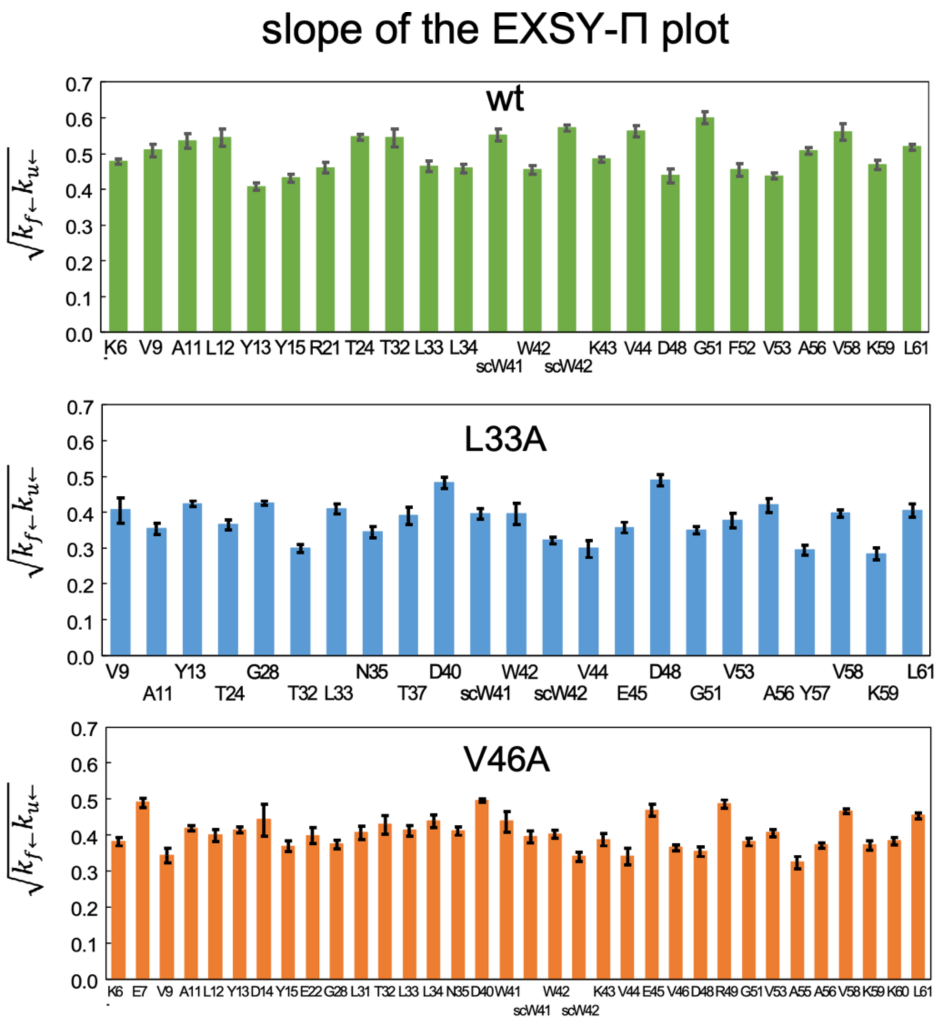

Supplementary Figure S9 Bar graphs showing $\sqrt{k_{f\leftarrow}k_{u\leftarrow}}$ (i.e., the slope of the EXSY-Π plot) of each residue. Error bars represent the standard error (SE) of the regression slope in the EXSY-Π plots.

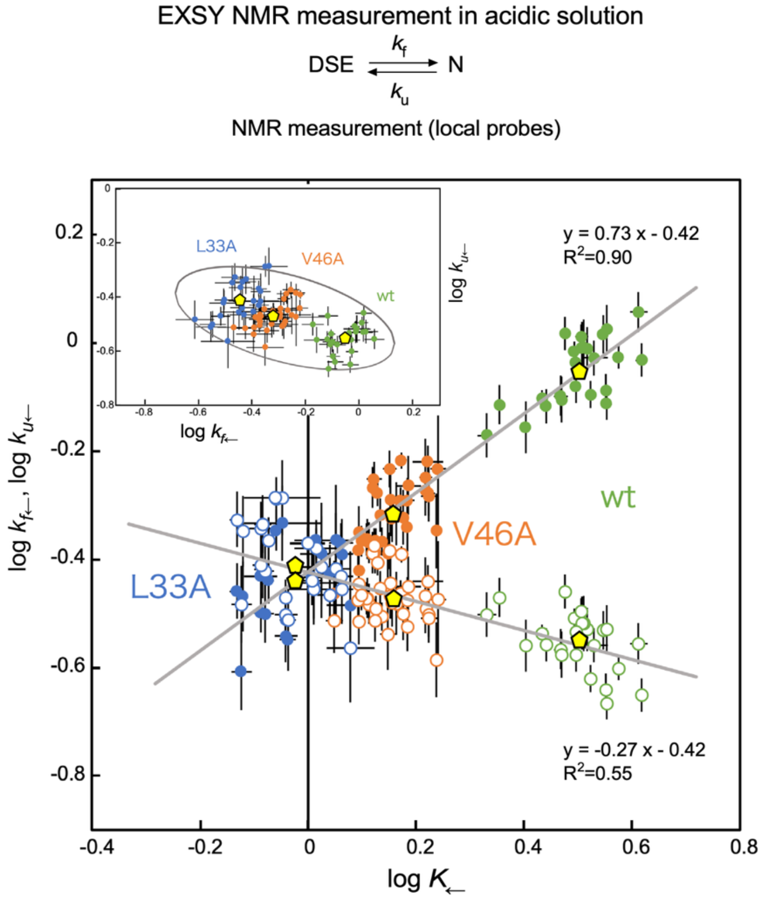

Supplementary Figure S10 Correlation plots between log $K_{\leftarrow}$, log $k_{f\leftarrow}$, and log $k_{u\leftarrow}$ for the validation of residue-based LFER. The plot is the same as in the main text (Fig. 3A) but with error bars (SE). The yellow pentagons show the centroids of the data point clusters. The inset shows the log $k_{u\leftarrow}$ vs. log $k_{f\leftarrow}$ plot with the 95% confidence ellipse.
